# A dual role of the conserved PEX19 helix in safeguarding peroxisomal membrane proteins

**DOI:** 10.1016/j.isci.2024.109537

**Published:** 2024-03-18

**Authors:** Jeonghyun Oh, Do Kyung Kim, Seung Hae Ahn, Ho Min Kim, Hyunju Cho

**Affiliations:** 1 Center for Biomolecular and Cellular Structure, Institute for Basic Science (IBS), Daejeon 34126, Republic of Korea; 2 Graduate School of Medical Science & Engineering, Korea Advanced Institute of Science and Technology (KAIST), Daejeon 34141, Republic of Korea

**Keywords:** Biological sciences, Molecular biology, Molecular interaction, Omics, Proteomics

## Abstract

Accurate localization of membrane proteins is essential for proper cellular functioning and the integrity of cellular membranes. Post-translational targeting of peroxisomal membrane proteins (PMPs) is mediated by the cytosolic chaperone PEX19 and its membrane receptor PEX3. However, the molecular mechanisms underlying PMP targeting are poorly understood. Here, using biochemical and mass spectrometry analysis, we find that a conserved PEX19 helix, αd, is critical to prevent improper exposure of the PEX26 transmembrane domain (TMD) to cytosolic chaperones. Furthermore, the αd helix of PEX19 interacts with the cytosolic domain of the PEX3 receptor, thereby triggering PEX26 release at the correct destination membrane. The peroxisome-deficient PEX3-G138E mutant completely abolishes this secondary interaction, leading to lack of PEX3-induced PEX26 release from PEX19. These findings elucidate a dual molecular mechanism that is essential to membrane protein protection and destination-specific release by a molecular chaperone.

## Highlights

• The αd helix of PEX19 prevents PEX26 loss to other cytosolic chaperones
• PEX3 activates PEX26 release from PEX19
• The αd helix of PEX19 serves as a secondary PEX3-binding site
• PEX19-αd binding to PEX3 is required for proper peroxisomal localization of PEX26

## Graphical abstract

**Figure:**
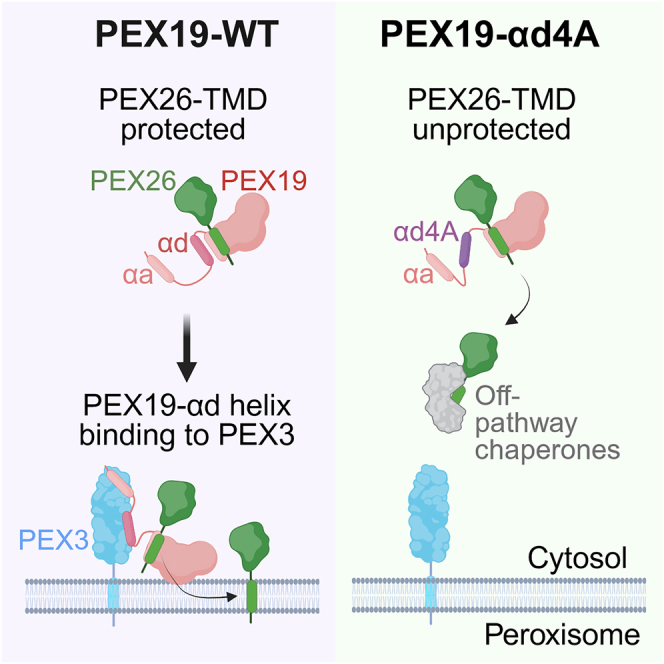

## Introduction

Membrane proteins represent ∼30% of the human proteome and play essential roles in the regulation of cellular functions, including transport of molecules, signal transduction, enzyme activity, and inter-organelle communication.^1^ Due to these roles in various physiological processes, proper membrane protein targeting to specific cellular compartments is crucial for overall cellular function and membrane integrity. Peroxisomes are small membrane-bounded organelles that provide a controlled environment for essential oxidative reactions and lipid metabolism.^2^^,^^3^^,^^4^ Membrane protein targeting to peroxisomes is critical for the formation of the appropriate membrane composition required for the biogenesis and function of mature peroxisomes.^2^^,^^5^^,^^6^ Indeed, aberrant membrane protein localization disrupts peroxisome biogenesis, eventually leading to impaired metabolism and developmental abnormalities.^2^^,^^5^

Direct targeting of peroxisomal membrane proteins (PMPs) is mediated by the cytosolic chaperone PEX19 (peroxisomal biogenesis factor 19) and its docking receptor PEX3 (peroxisomal biogenesis factor 3).^7^^,^^8^^,^^9^ Human PEX19 consists of an intrinsically disordered N-terminal domain (NTD) that serves as a PEX3-binding site and a folded C-terminal domain (CTD) where PMPs bind.^10^^,^^11^^,^^12^^,^^13^^,^^14^ PEX19 directly captures newly synthesized PMPs in the cytosol, thus preventing PMP aggregation.^15^^,^^16^ PMP-bound PEX19 is recruited to the peroxisomal membrane by the cytosolic domain of PEX3, which interacts with an N-terminal PEX19 domain, the αa helix.^11^^,^^12^^,^^17^ In addition, farnesylation of the C-terminal cysteine residue in the PEX19-CaaX motif increases the binding affinity of a PMP-derived peptide.^10^ Intriguingly, a previous study showed that mutation of four hydrophobic residues to alanine in the amphipathic αd helix located at the NTD of *Neurospora* PEX19 (PEX19-αd4A) impaired *Neurospora* PEX26 targeting to the peroxisome. However, the PEX19-αd helix is dispensable for chaperone activity and overall PEX3 binding.^15^ Thus, how the αd helix of PEX19 regulates PMP targeting and whether the NTD of human PEX19 has additional roles in PMP targeting beyond PEX3 interaction remain enigmatic.

The tail-anchored membrane proteins (TAs) are a classic example of PMPs that are targeted to the peroxisome through the PEX19/PEX3 pathway.^5^^,^^18^^,^^19^^,^^20^ TAs contain a single transmembrane domain (TMD) near the C-terminus.^21^ Due to this topology, TMDs of TAs are captured by cytosolic chaperones and targeted to their destined membrane post-translationally.^18^^,^^22^^,^^23^ During the post-translational targeting process, hydrophobic TMDs of TAs are at risk of promiscuous interactions with off-pathway chaperones. Therefore, TA-TMDs require specialized chaperones to shield them from other chaperones in the cytosol until they are safely inserted in the membrane.^24^^,^^25^^,^^26^ PEX26, a peroxisomal TA involved in the import and export of peroxisome matrix proteins,^27^^,^^28^ is known to be inserted into the peroxisome in a PEX19-dependent manner but is not bound by the ER TA chaperone, Get3/TRC40.^15^^,^^16^^,^^29^ However, how PEX19 protects TAs from such off-pathway chaperones is currently unknown.

Here, we use biochemical and *in vitro* PMP import assays to address how the peroxisomal TA PEX26 is properly targeted. Our results show that the αd helix at the PEX19-NTD prevents PEX26 loss to other chaperones. Furthermore, we find that the cytosolic domain of PEX3 interacts with the αd helix of PEX19, a secondary PEX3-binding site, thus activating PEX26 release from PEX19. The peroxisome-deficient mutant PEX3-G138E completely abolishes both the interaction with the αd helix of PEX19 and PEX3-activated PEX26 dissociation. Therefore, our study reveals that the PEX19-αd helix plays a dual role by shielding peroxisomal TAs from off-pathway chaperones and cooperating with PEX3 to promote TA release to the peroxisomal membrane.

## Results

### The αd helix of PEX19 prevents PEX26 loss to other chaperones

**Figure 1:**
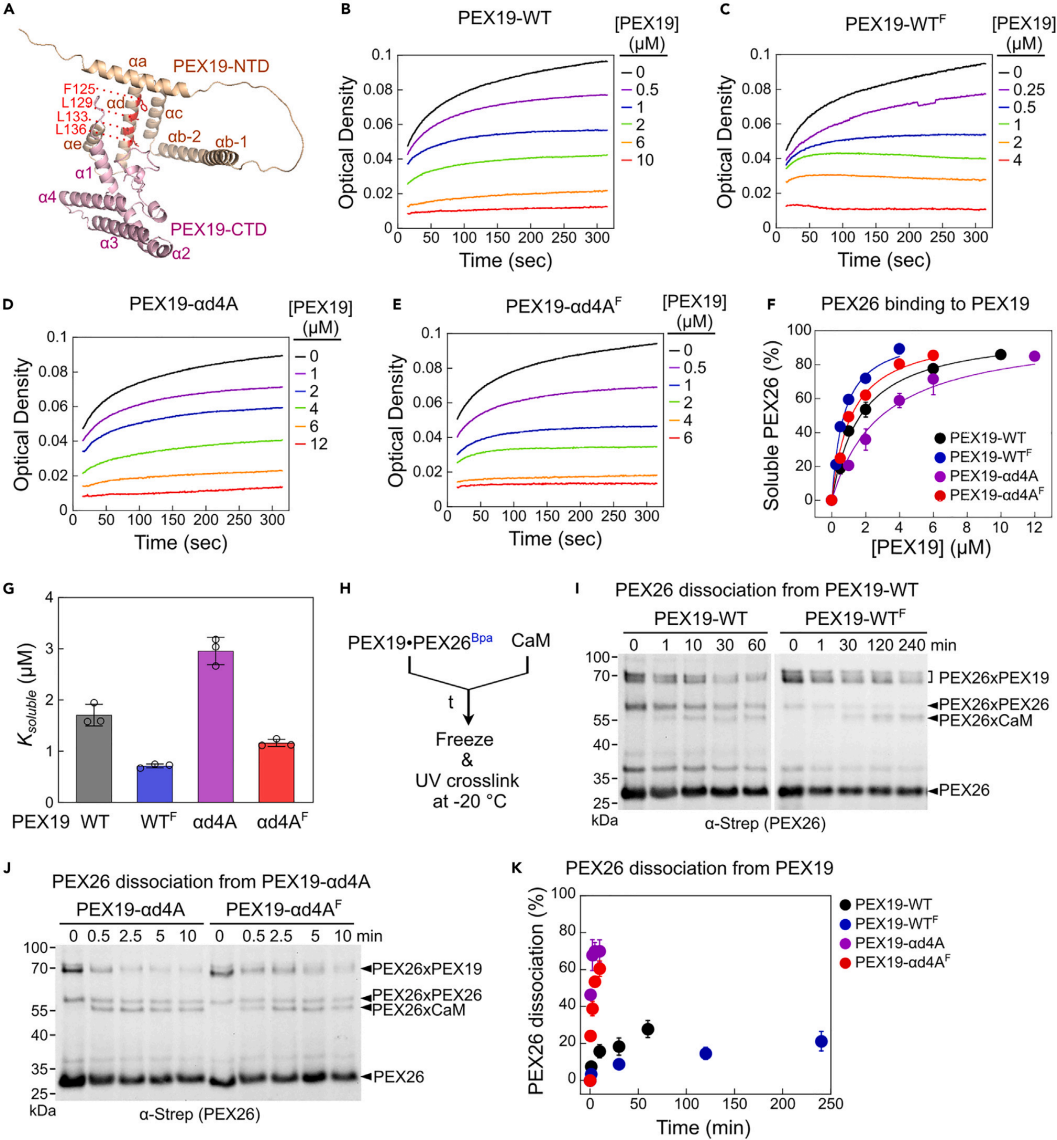
The αd helix of PEX19 is crucial for protecting the TMD of PEX26 from other chaperones (A) The AlphaFold-predicted structure of human PEX19.^30^^,^^31^ Four conserved hydrophobic residues (F125, L129, L133, L136) located within the αd helix of PEX19^15^ were highlighted in red (AF-P40855-F1). (B‒E) Time courses of PEX26 (1.5 μM) aggregation in the presence of indicated concentrations of PEX19-WT (B), farnesylated PEX19-WT^F^ (C), PEX19-αd4A (D), and farnesylated PEX19-αd4A^F^ (E). After mixing PEX26 with PEX19 proteins for 15 s, the real-time measurement of the optical density of PEX26 at 360 nm was conducted using a UV spectrophotometer. (F and G) PEX19 variants solubilize PEX26 in a dose-dependent manner. The data in (F) were fit to Equation 1, and the *K*_*soluble*_ values of PEX19 variants were summarized in (G). (H) Schematic representation of the Bpa crosslinking assay used to monitor PEX26^Bpa^ dissociation from PEX19 in the presence of a chaser chaperone, CaM; 20 μM CaM was mixed with PEX19·PEX26^Bpa^ to initiate PEX26 release. The zero time point samples were collected before the addition of CaM. At indicated times (t), aliquots (15 μL) of the reaction were frozen and analyzed by UV crosslinking at −20°C. (I and J) A representative western blot image of PEX26^Bpa^ dissociation from PEX19-WT (I) and PEX19-αd4A (J) proteins. The total volume (15 μL) of each sample at the indicated time was loaded onto 8% Tricine gels, and the non-crosslinked and crosslinked PEX26 proteins were probed with a Strep antibody. (K) Quantification of PEX26 dissociation from the Bpa crosslinking data in (I) and (J) and their replicates. PEX26 dissociation (%) was calculated as described in STAR methods. All values in (F), (G), and (K) are reported as mean ± SD, with n = 3. Error bars are shown but may not be visible in some cases.

The intrinsically disordered NTD of PEX19 contains several helices^15^ (Figure 1A). As previously shown,^15^ the hydrophobic residues of both the αa helix, a known PEX3-binding site, and the αd helix are highly conserved (Figure S1). To dissect which molecular steps in peroxisomal TA targeting require the conserved αd helix of human PEX19, we established a turbidity assay that monitors the chaperone activities of PEX19 proteins (Figures 1B–1G). We performed an *in vitro* farnesylation reaction using the yeast farnesyltransferases, Ram1 and Ram2^10^ (Figures S2A and S2B), and then purified the farnesylated PEX19-WT (denoted as PEX19-WT^F^) and PEX19-αd4A (PEX19-αd4A^F^; F125A, L129A, L133A, L136A) proteins (Figure 1A). The affinity-purified farnesylated PEX19 proteins exhibit increased electrophoretic mobility in SDS-PAGE compared with non-farnesylated PEX19^10^ (Figures S2B‒S2D). Irrespective of the farnesylation state, PEX19-WT and PEX19-αd4A had similar sensitivity to limited trypsin digestion (Figures S2E and S2F), suggesting that the αd4A mutation does not cause large structural alteration on the PEX19 protein. As a model PMP, we fused the N-terminal 2×Strep-tagged SUMO domain to the PEX26 targeting sequences (237–305 aa) encompassing the TMD and C-terminal charged tail of PEX26.^16^ The non-cleavable, soluble SUMO domain was previously used to purify detergent-solubilized ER and mitochondrial TAs.^26^^,^^32^^,^^33^ We purified the recombinant PEX26 protein solubilized in 0.05% LDAO (N,N-dimethyl-1-dodecanamine-N-oxide) (Figure S2G).

In the turbidity assay, dilution of detergent-purified PEX26 in aqueous buffer led to rapid PEX26 aggregation (Figure 1B, black). However, PEX19-WT and PEX19-αd4A proteins prevented PEX26 aggregation in a dose-dependent manner (Figures 1B–1E). The concentrations of non-farnesylated PEX19-WT and PEX19-αd4A required for half-maximal solubilization of PEX26 (*K*_*soluble*_) were 1.71 ± 0.21 μM (Figures 1F and 1G, black and dark gray; Table S1) and 2.96 ± 0.27 μM (Figures 1F and 1G, magenta; Table S1), respectively. In addition, *K*_*soluble*_ values for farnesylated PEX19-WT^F^ and PEX19-αd4A^F^ proteins were 0.72 ± 0.04 μM (Figures 1F and 1G, blue; Table S1) and 1.16 ± 0.07 μM (Figures 1F and 1G, red; Table S1), respectively. Therefore, although the αd4A mutation on PEX19 weakly reduced the chaperone activities of PEX19, the farnesylation of PEX19 proteins increased chaperone activity toward PEX26 over 2-fold compared with non-farnesylated PEX19 proteins.

To test whether the αd helix of PEX19 is crucial for the dissociation of PMPs after PMPs are loaded onto PEX19, we monitored the dissociation of PEX26 from PEX19 using the UV crosslinking assay (Figure 1H). We site-specifically incorporated p-benzoyl-l-phenylalanine (Bpa) into the TMD of PEX26 at the L264 residue using amber suppression^34^ (Figure S3A), then purified the PEX19-PEX26^Bpa^ complex (Figures S3B‒S3D). We chased the purified PEX19-PEX26^Bpa^ complex with a cytosolic chaperone calmodulin (CaM), which is known to effectively bind TAs as well as small secretory proteins.^24^^,^^25^^,^^26^^,^^33^^,^^35^^,^^36^ Adding excess CaM to the purified PEX19-PEX26^Bpa^ complex led to the release of PEX26^Bpa^ from the complex and its binding to CaM, which facilitated UV-activated Bpa crosslinking of PEX26. This approach allowed us to monitor the direct interaction of PEX26 with two distinct chaperones, PEX19 and CaM (Figures 1H–1J). To avoid the potential effects arising from non-specific interactions of the hydrophobic PEX26^Bpa^ with plastic surfaces in the tested samples, we focused exclusively on the relative distribution of crosslinked PEX26xPEX19 and PEX26xCaM bands within each sample.

The release of PEX26 from farnesylated PEX19-WT^F^ occurred at a slower rate compared with non-farnesylated PEX19-WT (Figures 1I and 1K, blue vs. black). Surprisingly, irrespective of the farnesylation state of PEX19, PEX26 rapidly dissociated from PEX19-αd4A. In the presence of CaM, 70% of PEX26 was released from PEX19-αd4A within 10 min, whereas only 16% of PEX26 dissociated from PEX19-WT in the same period (Figures 1J and 1K, black vs. magenta). This difference in dissociation kinetics was also observed in the presence of another cytosolic chaperone, SGTA, known to bind to ER TAs^25^ (Figures S3E and S3F). In the presence of SGTA, approximately 50% of PEX26 was released from PEX19-αd4A within 20 min. In contrast, PEX26 was scarcely released from PEX19-WT even after 80 min (Figures S3E and S3F). Furthermore, the observed dissociation rates of PEX26 from PEX19-αd4A were strongly accelerated by increasing the CaM concentrations, whereas PEX26 release from PEX19-WT appeared to remain similar (Figures S4A and S4B vs. S4C-S4E). Consistent with a previous study,^24^^,^^26^ this linear dependency of the chase concentrations on the observed PEX26 dissociation rates for the PEX19-αd4A-PEX26 complex indicates that CaM actively invades and releases PEX26 from this complex (Figures S4C‒S4E). In contrast, the unaltered dissociation kinetics of PEX26 in the presence of two different concentrations of CaM suggests that CaM is more likely to function as a passive trap, binding and sequestering released PEX26 from PEX19-WT (Figures S4A and S4B). Taken together, our PEX26^Bpa^ crosslinking data suggest that the αd helix of PEX19 is crucial in preventing the promiscuous PEX26 handoff from PEX19 to other cytosolic chaperones.

### The cytosolic domain of PEX3 activates PEX26 release from PEX19

**Figure 2:**
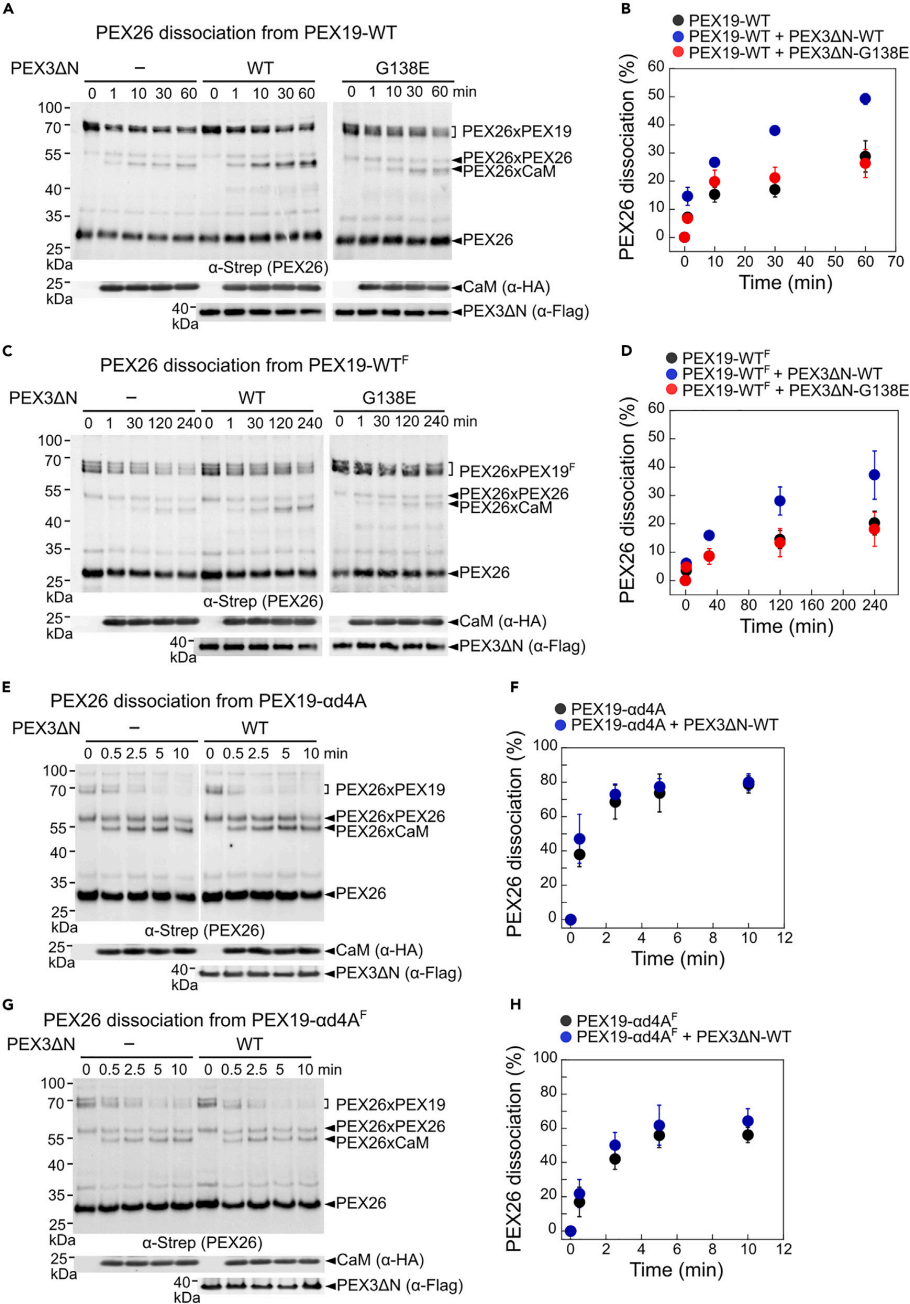
The cytosolic domain of PEX3-WT activates PEX26 dissociation from PEX19-WT, whereas PEX3-G138E does not (A‒D) (A and C) Representative western blot images of PEX26^Bpa^ dissociation from PEX19-WT (A) and PEX19-WT^F^ (C). In the absence and presence of 400 nM PEX3ΔN variants, PEX19-WT·PEX26^Bpa^ and PEX19-WT^F^·PEX26^Bpa^ complexes at the final concentrations of 400 nM PEX19 proteins were incubated with 20 μM CaM. The zero time point samples were collected before the addition of CaM to the PEX19·PEX26^Bp^^a^. At the indicated time points, aliquots of the reactions were frozen and analyzed by UV crosslinking. PEX26, PEX3ΔN, and CaM were probed in immunoblots with Strep, Flag, and HA antibodies, respectively. (B and D) Quantification of PEX26 dissociation from the Bpa crosslinking data in (A) and (C) and their replicates. (E‒H) (E and G) Representative western blot images of PEX26^Bpa^ dissociation from PEX19-αd4A (E) and PEX19-αd4A^F^ (G). PEX26^Bpa^ dissociation assays from PEX19-αd4A (E) and PEX19- αd4A^F^ (G) were conducted in the same manner as in (A) and (C). (F and H) Quantification of PEX26 dissociation from the Bpa crosslinking data in (E) and (G) and their replicates. All values in (B), (D), (F), and (H) are reported as mean ± SD, with n = 3. Error bars are shown but may not be visible in some cases.

The cytosolic domain of PEX3 interacts with the αa helix of PEX19, thereby facilitating the recruitment of PEX19 to the peroxisomal membrane.^11^^,^^12^ Furthermore, a previous study showed that two hydrophobic residues (L209 and L261) at the base of the *Neurospora crassa* PEX3-α2 and α3 helices are required for PEX26 insertion into the peroxisome.^15^ This study suggested that the cytosolic domain of PEX3 plays an additional role in PMP targeting beyond its interaction with the αa helix of PEX19. Using the PEX26^Bpa^ crosslinking assay established in Figure 1H, we measured PEX26 release from PEX19 in the presence of the cytosolic domains of PEX3-WT and the PEX3-G138E mutant, which is incapable of forming peroxisomes in peroxisome-deficient CHO cells (ZPG208).^37^^,^^38^ The cytosolic domain of PEX3-WT (PEX3ΔN-WT, 41-373aa), lacking its N-terminal TMD, induced significantly greater PEX26 release from both non-farnesylated PEX19 and farnesylated PEX19 (Figures 2A–2D, black vs. blue). In contrast, the peroxisome-deficient PEX3 mutant, PEX3ΔN-G138E,^38^ was not able to activate PEX26 release from PEX19-WT proteins (Figures 2A–2D, black vs. red). Furthermore, in the case of both the PEX19-αd4A-PEX26 and PEX19-αd4A^F^-PEX26 complexes, PEX3ΔN-WT did not alter PEX26 dissociation from PEX19-αd4A proteins (Figures 2E–2H). Together, these data suggest that the cytosolic domain of PEX3 triggers PEX26 release from PEX19, possibly a prerequisite for PMP insertion into the peroxisomal membrane.

### The αd helix of PEX19 directly interacts with the cytosolic domain of PEX3

We next considered why PEX19-αd4A and PEX3ΔN-G138E mutants cannot support PEX3-induced PEX26 release. The simplest explanation is that these mutants disrupt intermolecular interactions between PEX19 and PEX3. We tested this possibility by performing His_6_-PEX19 pull-down experiments at the same concentrations of PEX19 and PEX3 used in the PEX26^Bpa^ crosslinking experiments (Figure 2). We found that half of the peroxisome-deficient PEX3ΔN-G138E mutant binds PEX19-WT (Figures S5A and S5B). As expected, only ∼17% of PEX3ΔN-W104A, a PEX19-binding deficient mutant,^11^^,^^39^ was copurified with PEX19-WT (Figures S5A and S5B). In contrast, PEX3ΔN-WT bound the mutated PEX19-αd4A protein at levels comparable to PEX19-WT (Figures S5A and S5B). Therefore, although PEX19-αd4A lacks the PEX3-induced PMP release, it does not account for overall binding to the PEX3 receptor.

**Figure 3:**
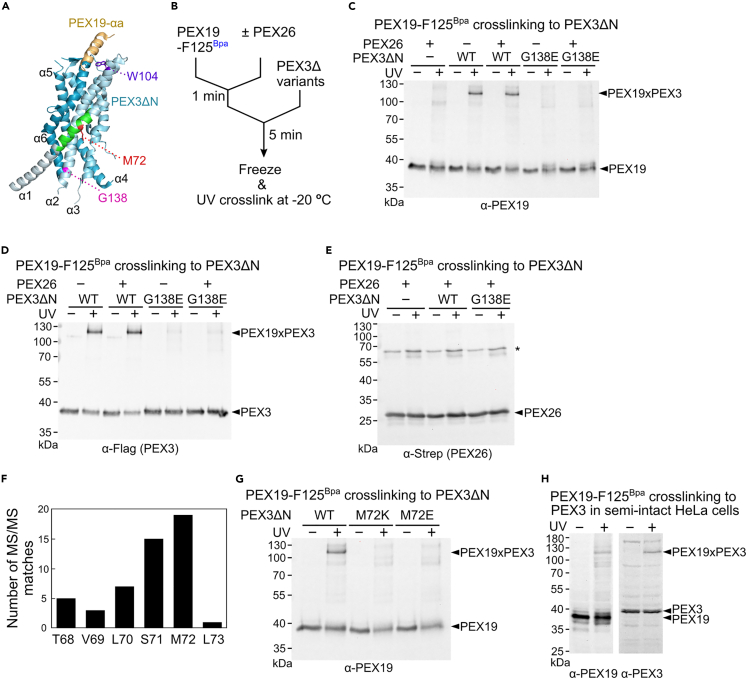
The αd helix of PEX19 interacts with the α1 of PEX3 (A) Crystal structure of PEX19-αa-bound PEX3ΔN (PDB 3MK4).^12^ The amino acid residues in the PEX19-F125^Bpa^-crosslinked PEX3 peptide identified from MS analysis were highlighted in green. The identified PEX19-F125^Bpa^-binding residue M72 in PEX3ΔN-α1 was shown in red. The known PEX19-αa-binding residue W104 and the disease-causing residue G138 (G138E) in PEX3ΔN-α2 were shown in purple and magenta, respectively. (B) A schematic representation of PEX19-F125^Bpa^ crosslinking to PEX3ΔN variants; 400 nM PEX19-F125^Bpa^ was incubated in the absence and presence of 100 nM PEX26 at room temperature for 1 min; 400 nM PEX3ΔN-WT and its variants were added to the reaction and further incubated at room temperature for 5 min. The frozen reaction samples were subjected to UV crosslinking. (C‒E) Western blot analysis of PEX19-F125^Bpa^ crosslinking to PEX3ΔN variants. The crosslinked proteins were probed using antibodies against PEX19, Flag (PEX3ΔN), and Strep (PEX26). “∗” represents the SDS-resistant PEX26 dimers in (E). (F) The number of matched peptides for PEX19-F125^Bpa^-PEX3 crosslink identified from MS analysis. The M72 residue in the PEX3 peptide (^64^TCNMTVLSMLPTLR^77^) was most frequently crosslinked with F125-Bpa in the PEX19 peptide (^115^VGSDMTSQQEBpaTSCLK^130^) (also see Figure S5H). (G) Western blot analysis of PEX19-F125^Bpa^ crosslinking to PEX3ΔN-WT, PEX3ΔN-M72K, and PEX3ΔN-M72E. The reactions were carried out in the same way as in (B). (H) Western blot analysis of PEX19-F125^Bpa^ crosslinking to the endogenous PEX3 in semi-intact HeLa cells; 200-nM PEX19-F125^Bpa^ was incubated with semi-permeabilized HeLa cells at room temperature for 1 h, after which the cell lysates were subjected to UV crosslinking as described in STAR methods.

A previous study found that the binding affinity of PEX19-αa peptide to the cytosolic domain of PEX3-WT (*K*_D_ = 40.8 nM) is 12-fold lower than that of the full-length PEX19 (*K*_D_ = 3.4 nM),^11^ suggesting that PEX19 contains a second PEX3-binding site that weakly contributes to the overall binding of PEX19 to PEX3 (Figure 3A). We hypothesized that the αd helix of PEX19 provides a secondary PEX3-binding site that induces PEX26 release from PEX19. To test this hypothesis, we genetically incorporated Bpa at the four different hydrophobic residues (F125, L129, L133, L136) located at the αd helix of PEX19 (Figure 1A) and monitored the interaction between PEX19 and PEX3ΔN-WT using the PEX19-F125^Bpa^ crosslinking assay (Figures 3B, S5C, and S5D). Of those four residues, only the F125^Bpa^ residue of PEX19 forms a ∼115 kDa crosslinked protein with PEX3ΔN-WT (Figure S5C). The intermolecular crosslink between PEX19-F125^Bpa^ and PEX3ΔN-WT formed even after pre-incubation with PEX26, indicating that PEX3ΔN-WT directly interacts with the αd helix of PEX19 (Figures 3C and 3D). In contrast, no PEX19-F125^Bpa^-mediated crosslink was formed in the presence of PEX3ΔN-G138E, suggesting that PEX3ΔN-G138E does not interact with the PEX19-αd helix (Figures 3C and 3D). Given that PEX19-αd4A and PEX19-WT bind to PEX3ΔN-WT equally well (Figures S5A and S5B), the αd4A mutation in PEX19 likely only disrupts the secondary interaction between the αd helix of PEX19 and PEX3ΔN-WT. In this case, the primary interaction of PEX19-αa helix with PEX3ΔN-WT would remain intact. In contrast, PEX3ΔN-G138E interferes with both the primary interaction with PEX19-αa and the secondary interaction with PEX19-αd (Figures S5A and S5B).

Several experimental data suggest that the αd helix of PEX19 is unlikely to directly interact with PEX26. None of the four hydrophobic residues in the PEX19-αd helix showed a distinct crosslinked band with PEX26 in the absence of PEX3 (Figure S5D). In addition, PEX19-F125^Bpa^ did not crosslink with PEX26 in the absence or presence of PEX3ΔN variants (Figures 3C and 3E). In contrast, PEX19-M179^Bpa^ located in the known PMP-binding α1 helix showed a distinct PEX19-PEX26 crosslinked band (Figures S5E and S5F). Considering the data in Figures 1, S3, and S4, it becomes evident that, although the hydrophobic residues in the αd helix of PEX19 do not directly bind to PEX26, the αd helix of PEX19 could be located proximally to the TMD of PEX26, thereby shielding it from other chaperones.

To further identify the PEX19-F125-binding site on the cytosolic domain of PEX3, we utilized a Bpa-crosslinking-based mass spectrometry approach.^40^^,^^41^ The PEX19-F125^Bpa^-PEX3ΔN-WT crosslink was in-gel digested with trypsin and AspN endoproteinases, and the cleaved peptides were further analyzed by LC-MS/MS (Figures S5G and S5H). Using a database search algorithm pLink 2,^42^ we identified the most frequently detected Bpa crosslinked peptide (PEX3 residues T64-R77), which represents over 90% of the identified crosslinked peptides (Figure 3A, highlighted with green). These residues are located in the α1 helix of PEX3, distinct from the primary PEX19-αa-binding site (Figure 3A).

Because mutating all four hydrophobic amino acids in the αd helix of PEX19 to alanine (PEX19-αd4A) abolishes PEX3-activated PEX26 release (Figures 2E–2H), the hydrophobic residues at the α1 helix of PEX3 may interact with the PEX19-F125 residue. Consistent with this hypothesis, mass spectrometry analysis showed that the hydrophobic M72 residue at the PEX3-α1 helix is the most frequently observed residue that crosslinks to PEX19-F125^Bpa^ (Figure 3F). Charge mutations of the M72 residue, PEX3ΔN-M72K and PEX3ΔN-M72E, failed to crosslink to the PEX19-F125^Bpa^ residue (Figure 3G), suggesting that the interaction between PEX3-α1 and PEX19-αd is dominated by inter-helical hydrophobic interactions. In contrast, the overall binding of PEX3ΔN-M72K to PEX19-WT is comparable with the wild-type protein, PEX3ΔN-WT (Figures S5A and S5B).

Next, we tested whether this secondary interaction between PEX19 and PEX3 occurs in cells. After washing out the cytosolic fraction (Figure S6A, steps I and II), the purified PEX19-F125^Bpa^ protein was incubated with the semi-permeabilized HeLa cells. The data of UV crosslinking experiment show that PEX19-F125^Bpa^ forms a ∼120 kDa crosslink to the full-length PEX3 protein in the cells (Figure 3H). This observation indicates that the membrane-embedded full-length PEX3 also interacts with the αd helix of PEX19. Collectively, our data suggest that the αd helix of PEX19 serves as a previously obscure secondary PEX3-binding site that mediates substrate dissociation once the TA reaches the peroxisome.

### Both αa and αd helices of PEX19-NTD are crucial for the peroxisomal targeting of PEX26

**Figure 4:**
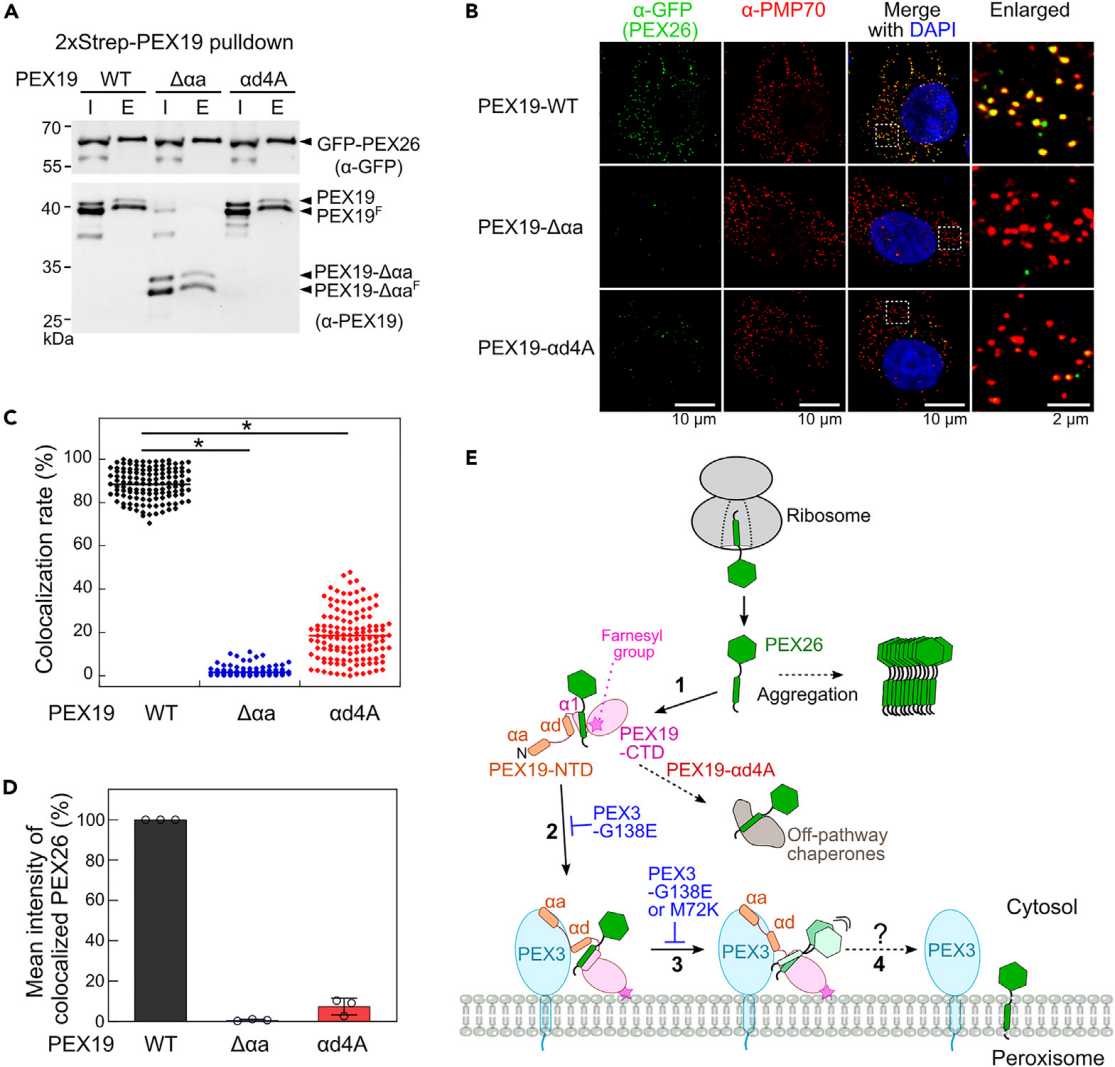
PEX19-αd4A significantly reduced the peroxisomal targeting of PEX26 (A) 2×Strep-PEX19 pull-down experiments. 2×Strep-PEX19 variants (WT, Δ αa, and αd4A) and GFP-PEX26 were co-expressed in HEK293T cells, and the cytosolic fractions were subjected to 2×Strep-PEX19 pull-down experiments. I and E denote input and elution, respectively. The I and E fractions were subjected to SDS-PAGE and western blotting with antibodies against Strep and GFP. (B) The 2×Strep-PEX19·GFP-PEX26 complexes from the E fractions (A) were used for PEX26 targeting experiments in semi-permeabilized HeLa cells, as shown in Figure S4A. After incubating the 2×Strep-PEX19·GFP-PEX26 complexes for 1 h, the cells were fixed and further analyzed by immunofluorescence (scale bar: 10 μm); 5.3-fold enlarged images of the boxed areas were shown separately in the right panel (scale bar: 2 μm). (C) Colocalization rates of PEX26 with the peroxisomal membrane protein (PMP70). A total of 130 cells from three biological replicates were analyzed using LAS X software. The lines indicate the mean values of the colocalization rate of PEX26 for each condition (n = 130). ∗p < 0.0001 (Student’s t test). (D) Mean intensity of colocalized PEX26 with PMP70. The mean intensities of peroxisome-localized PEX26 in (C) were analyzed using LAS X software. Values are reported as mean ± SD, with n = 3 (three biological replicates). Error bars are shown but may not be visible in some cases. (E) A proposed model of PEX26 targeting to the peroxisome. PEX19 rapidly captures free PEX26 in the cytosol (step 1). As shown in (A), a majority of PEX19 proteins in the cytosol are farnesylated (asterisk). Farnesylated PEX19 displays an increased binding affinity to PEX26. The αd helix of PEX19 protects against PEX26 loss to off-pathway chaperones (step 2). The αa helix of PEX19 primarily interacts with the cytosolic domain of PEX3. The secondary interaction of PEX19-αd helix with PEX3 destabilizes the PEX19·PEX26 complex, thereby inducing the release of PEX29 to the membrane and further leading to its membrane insertion (steps 3–4).

To check whether the secondary PEX3-binding site of PEX19 is important for the peroxisomal targeting of PEX26, we employed the established *in vitro* PMP import assay with semi-permeabilized cells^16^^,^^17^ (Figure S6A). We coexpressed 2×Strep-PEX19 variants and GFP-PEX26 (1–305 aa) in HEK293 cells and prepared 2×Strep-PEX19·GFP-PEX26 complexes from the cytosolic fraction of these cells (Figure 4A). While PEX19-Δαa (45–299 aa) removes the primary PEX3-binding site,^11^^,^^12^ PEX19-αd4A alters the secondary PEX3-binding site. After washing out ∼2/3 of cytosolic proteins, the freshly prepared 2×Strep-PEX19·GFP-PEX26 complexes were incubated with the semi-permeabilized HeLa cells (Figures S6A and S6B).

Incubation with the 2×Strep-PEX19-WT·GFP-PEX26 complex for 1 h led to an 88.4% PEX26 colocalization rate with PMP70, a peroxisomal maker protein (Figures 4B and 4C). In contrast, the shorter incubation (10 min) with the 2×Strep-PEX19-WT·GFP-PEX26 complex was insufficient for the stable targeting of PEX26 into peroxisome (Figures S6C‒S6E, blue). Furthermore, when 2×Strep-PEX19-Δαa·GFP-PEX26 and 2×Strep-PEX19-αd4A·GFP-PEX26 complexes were incubated with the semi-permeabilized cells for 1 h, PEX19-Δαa and PEX19-αd4A strongly inhibited the PEX26 localization into the peroxisome (Figures 4B and 4C). Similarly, the mean intensities of peroxisome-localized PEX26 were also strongly reduced in both the PEX19-Δαa and PEX19-αd4A mutants (Figures 4B and 4D). These different localization efficiencies of PEX26 to peroxisome are unlikely due to different amounts of PEX26 binding to the PEX19 variants (Figure 4A). Pre-incubation of 2×Strep-PEX19-WT·GFP-PEX26 complex with excess PEX3ΔN-WT almost completely abolished the colocalization of PEX26 with PMP70, further supporting the essential role of PEX19-PEX3 interactions in the peroxisomal targeting of PMPs (Figures S6C‒S6E). Together, these data highlight the dual role of the PEX19-αd helix in protecting PEX26 from other chaperones and interacting with the cytosolic domain of PEX3 to ensure the efficient localization of PEX26 into the peroxisome.

## Discussion

Using biochemical assays and mass spectrometry analysis, we revealed that the αd helix of PEX19 serves as a release modulator for PMPs at the peroxisome. PEX19 rapidly captures PEX26 upon its release from the ribosome (step 1),^15^ and farnesylation of PEX19 at the C-terminus enhances PEX26 solubility. The αd helix of PEX19 is essential in preventing PEX26 loss to off-pathway chaperones. Removal of conserved hydrophobic residues in the αd helix of PEX19 (PEX19-αd4A) led to premature PEX26 release to off-pathway chaperones. PEX26-bound PEX19 is recruited to PEX3 via the primary PEX3 binding helix, PEX19-αa (step 2).^11^^,^^12^ The secondary PEX3-binding site, PEX19-αd, further interacts with the cytosolic domain of PEX3, thereby activating PEX26 release to the membrane (step 3). The peroxisome-deficient mutants PEX3-G138E and PEX3-M72K lack this secondary interaction with the αd of PEX19. Finally, PEX26 is inserted into the membrane via unknown mechanisms (step 4).

Despite the functional importance of PEX19 farnesylation *in vivo*, only a few previous studies showed that farnesylation of PEX19 enhances PMP-binding affinity.^10^^,^^43^ Using a turbidity assay that monitors TA aggregation, we showed that farnesylated PEX19-WT^F^ captures and solubilizes PEX26 more efficiently than non-farnesylated PEX19-WT (Figures 1B–1G). The PEX26 solubilization constant for PEX19-WT^F^ is about 2-fold lower than PEX19-WT (Figures 1F and 1G). This mild enhancement of PEX19 chaperone activity by farnesylation is consistent with a previous study of *Neurospora* PEX19, which qualitatively assessed chaperone activity using ultracentrifugation.^15^ Furthermore, the required time to release ∼20% PEX26 from PEX19-WT and PEX19-WT^F^ to the external chaperone, CaM, is 30∼40 min and 240 min, respectively (Figures 1I–1K). Thus, farnesylation of PEX19 not only helps to capture PMPs more effectively but also prevents TA loss from PEX19 to other cytosolic chaperones.

Membrane protein chaperones appear to possess structural features that protect the hydrophobic TMDs of membrane proteins from off-pathway chaperones in the crowded cytosolic environment. Our mechanistic dissections revealed that the αd helix of PEX19-NTD minimizes the loss of PEX26 from PEX19 to external chaperones, such as CaM and SGTA. In the presence of CaM, 60% to 80% of PEX26 was released from PEX19-αd4A proteins within 5 min, whereas only 20% to 30% of PEX26 dissociated from PEX19-WT proteins within 4 h (Figures 1 and 2). PEX19-αd4A lost over 40% of PEX26 to SGTA within 10 min, whereas only less than 4% of PEX26 was released from PEX19 within 80 min (Figures S3E and S3F). Previous studies showed that the α8 helix of the yeast cytosolic chaperone Get3 serves as a lid for the ER TA-binding groove, protecting ER TAs from other cytosolic chaperones.^24^^,^^44^^,^^45^ Similarly, although AlphaFold^30^^,^^31^ predicts that the intrinsically disordered NTD of PEX19 forms an open conformation (Figure 1A), the αd helix may adopt a more compact conformation toward the α1 helix upon TA binding to the PEX19-CTD, thereby shielding the hydrophobic TMDs of peroxisomal TAs.^10^^,^^15^^,^^46^ Given the lack of structural information on the PEX19-NTD, this hypothesis would require future experimental testing.

The mechanism activating cargo release from TA-loaded PEX19 to the membrane remains enigmatic. Our results suggest that the secondary interaction between PEX19-αd and PEX3-α1 modulates the dissociation of PEX26 (Figures 2 and 3). Although the PEX19-αd4A mutant binds to the cytosolic domain of PEX3 (Figures S5A and S5B), it loses PEX3-activated PEX26 release, potentially due to lack of the secondary interaction between PEX19-αd and PEX3-α1. Furthermore, the PEX3-G138E and M72K mutants,^38^^,^^47^ which lead to lack of peroxisomes, completely abolished this secondary interaction between PEX19-αd and PEX3-α1 (Figures 3C and 3G). Together with previous studies,^11^^,^^12^ these data indicate that the secondary interaction of PEX19-αd with PEX3ΔN destabilizes the PEX19-TA complex prior to membrane insertion (Figure 2). However, high-affinity binding between PEX19 and PEX3ΔN is primarily mediated by the N-terminal PEX19-α1 helix.^11^^,^^12^ An analogous mechanism was previously observed in TA targeting to the ER membrane.^48^ In the guided entry of tail-anchored protein (GET) pathway, the Get2 N-terminal helices mediate the primary interaction between the cytosolic domain of Get2 and Get3.^49^^,^^50^^,^^51^ However, a secondary interaction between the internal helices of Get2 and Get3 remodels the latter protein to optimize TA release.^48^ Therefore, multiple interactions between chaperones and their membrane receptor proteins may ensure efficient recruitment of the targeting complex as well as destination-specific release of membrane proteins to their target membranes.

In conclusion, our study revealed a dual role for PEX19-αd in shielding the TMD of TAs from other cytosolic chaperones and in interacting with PEX3 to trigger TA release prior to membrane insertion. In contrast to other chaperones that use ATP hydrolysis and cochaperones to regulate their conformations, PEX19 appears to employ a conserved helix, αd, at its intrinsically disordered NTD for TA targeting. This switch-like αd helix of PEX19 would minimize the exposure of the hydrophobic TMD to other proteins and only allow TA release upon binding to the cytosolic domain of PEX3. Given that PEX19 is known to deliver TA proteins to mitochondria,^19^^,^^52^ we envision that PEX19 uses a similar mechanism for targeting of mitochondrial TA proteins. Guided by this study, future structural and single-molecule analyses could provide mechanistic insight into how an ATP-independent chaperone dynamically modulates its conformation for proper membrane protein targeting.

### Limitations of the study

As a model substrate for PEX19, we used the peroxisomal TA, PEX26, which contains only one TMD near the C-terminus. Given that PEX19 is also known to regulate the peroxisomal targeting of multi-spanning PMPs,^7^^,^^9^^,^^17^ it remains an open question whether the αd helix of PEX19 plays the same dual role in protecting multiple TMDs from other cytosolic chaperones and assisting PMP release to the peroxisomal membrane.

The PMP import assay used in this study contains a significant amount of cytosolic fractions. Although our results suggest that the αd helix of PEX19 is crucial for the peroxisomal localization of TAs, the assay would not be optimal to dissect two molecular steps: PEX26 dissociation before and after PEX19 binding to PEX3. Currently, there is no solid evidence indicating whether PEX19-PEX3 constitutes a minimal targeting complex for peroxisomal TAs or if an additional targeting factor is necessary. Once this information is revealed, biochemical reconstitution of the targeting complex into proteoliposomes will help further elucidate the molecular mechanism underlying the insertion of TAs into the peroxisome (steps 3–4 in Figure 4E).

## STAR★Methods

### Key resources table

**Table:** 

REAGENT or RESOURCE	SOURCE	IDENTIFIER
**Antibodies**		

THE™ NWSHPQFEK Tag antibody (anti-Strep)	Genscript	Cat#A01732; RRID: AB_2622218
DYKDDDDK Tag antibody (5A8E5)	Genscript	Cat#A00187; RRID: AB_1720813
IRDye800CW Goat anti-Mouse IgG secondary antibody	Licor	Cat#926–32210; RRID: AB_621842
IRDye800CW Goat anti-Rabbit IgG secondary antibody	Licor	Cat#926–32211; RRID: AB_621843
GFP antibody	Abcam	Cat#ab13970; RRID: AB_300798
PMP70 antibody	Invitrogen	Cat#PA1-650; RRID: AB_2219912
Goat anti-Rabbit IgG (H + L) Cross-Adsorbed Secondary Antibody, Alexa Fluor™ 568	Invitrogen	Cat#A-11011; RRID: AB_143157
Alexa FluorJa 647 AffiniPure™ F(ab')₂ Fragment Donkey Anti-Chicken IgY (IgG) (H + L)	Jackson ImmunoResearch	Cat#703-606-155; RRID: AB_2340380
PEX19 Antibody	Novus Biologicals	Cat#NBP2-43757; RRID: AB_3086736
PEX3 Antibody	Novus Biologicals	Cat#NBP3-18138; RRID: AB_3086737

**Bacterial and virus strains**		

DH5α	Enzynomics	Cat#CP011
BL21 Star^TM^ (DE3)	Invitrogen	Cat#C601003

**Chemicals, peptides, and recombinant proteins**		

Benzonase® Nuclease	Sigma-Aldrich	Cat#E1014
cOmplete^TM^ EDTA-free protease inhibitor cocktail	Roche	Cat#11836170001
n-Dodecyl-N,N-Dimethylamine-N-Oxide (LDAO)	Anatrace	Cat#D360
CelLytic^TM^ B Cell Lysis Reagent	Sigma-Aldrich	Cat#C8740
*d*-Desthiobiotin	Sigma-Aldrich	Cat#D1411
L-(+)-Arabinose	Sigma-Aldrich	Cat#A3256
H-p-Bz-Phe-OH (Bpa)	Bachem	Cat#4017646
Farnesyl pyrophosphate ammonium salt	Sigma-Aldrich	Cat#F6892
SimplyBlue™ SafeStain	Invitrogen	Cat#LC6065
Trypsin	Sigma-Aldrich	Cat#T6567
Endoproteinase Asp-N Sequencing Grade	Roche	Cat#11054589001
Lipofectamine™ 3000 Transfection Reagent	Invitrogen	Cat#L3000
Digitonin	Sigma-Aldrich	Cat#D5628
Halt™ Protease Inhibitor Cocktail, EDTA-Free (100X)	Thermo Fisher Scientific	Cat#87785
DAPI	Invitrogen	Cat#D1306
Dulbecco’s Modified Eagle Medium, GlutaMAX^TM^	Gibco	Cat#10569044
Fetal Bovine Serum	Gibco	Cat#16140071
Penicillin-Streptomycin	Gibco	Cat#15140122
Strep-Tactin® Sepharose® resin	IBA Lifesciences	Cat#2-1201
Ni-NTA Agarose	Qiagen	Cat#30230
TALON Metal Affinity Resin	TaKaRa	Cat#635502

**Deposited data**		

Mass spectrometry raw data	This paper	Mendeley dataset: https://doi.org/10.17632/dsrjx52zty.2

**Experimental models: Cell lines**		

HeLa (Human cervical cancer cell line)	ATCC	RRID: CVCL_0030
HEK293T (Human embryonic kidney cell line)	ATCC	RRID: CVCL_0063

**Oligonucleotides**		

Primers for PCR: PEX19 αd4A mutation forward: 5′-CTTGCGCAAAGGAAACAG CGAGTGGAGCAGCCAAAAATGCCACT GACCTTCAGAAC-3′	This paper	N/A
Primers for PCR: PEX19 αd4A mutation reverse: 5′-CTCGCTGTTTCCTTTGCGCAAGAAGTTGCT TCTTGTTGGGAGGTCATATCACTGCC-3′	This paper	N/A
Primers for PCR: PEX3 W104A mutation forward: 5′-CAAGCTAGAAATAGCGGAGGATCTGAAGA TAATAAGTTTCACAAGAAGTAC -3′	This paper	N/A
Primers for PCR: PEX3 W104A mutation reverse: 5′-CTTCAGATCCTCCGCTATTTCTAGCTTGTTT GAAGGCCTGTTTTTTAGC-3′	This paper	N/A
Primers for PCR: PEX3 G138E mutation forward: 5′-AACATAATTGGTGAATATATTTACCTGGAT AATGCAGCAGTTGGC-3′	This paper	N/A
Primers for PCR: PEX3 G138E mutation reverse: 5′-CAGGTAAATATATTCACCAATTATGTTTAA CTGGACCCGCAAAAG-3′	This paper	N/A
Primers for PCR: PEX3 M72K mutation forward: 5′-GACAGTGCTGTCCAAACTTCCAACACTGA GAGAGGCCTTAATG-3′	This paper	N/A
Primers for PCR: PEX3 M72K mutation reverse: 5′-GTGTTGGAAGTTTGGACAGCACTGTCATA TTGCAAGTCC-3′	This paper	N/A
Primers for PCR: PEX3 M72E mutation forward: 5′-GACAGTGCTGTCCGAACTTCCAACACTGA GAGAGGCCTTAATG-3′	This paper	N/A
Primers for PCR: PEX3 M72E mutation reverse: 5′-GTGTTGGAAGTTCGGACAGCACTGTCA TATTGCAAGTCC-3′	This paper	N/A

**Recombinant DNA**		

pET33b -His_6_-TEV-PEX19-WT (1–299 aa)	This paper (pHC24)	N/A
pET33b -His_6_-TEV-PEX19-αd4A (F125A, L129A, L133A, L136A)	This paper (pHC184)	N/A
pET33b -His_6_-SUMO-PEX19-F125^Amb^	This paper (pHC197)	N/A
pET33b -His_6_-SUMO-PEX19-L129^Amb^	This paper (pHC198)	N/A
pET33b -His_6_-SUMO-PEX19-L133^Amb^	This paper (pHC199)	N/A
pET33b -His_6_-SUMO-PEX19-L136^Amb^	This paper (pHC200)	N/A
pET33b -His_6_-SUMO-PEX19-M179^Amb^	This paper (pHC259)	N/A
SUMO-Protease (ulp1 403-621aa)-His_6_	Cho. et al.^32^	N/A
pREST-HA-CaM-TEV-His	Shao et al.^36^	N/A
pET33b -His_6_-SUMO-Flag-PEX3ΔN-WT (41-373aa)	This paper (pHC39)	N/A
pET33b -His_6_-SUMO-Flag-PEX3ΔN-M72K	This paper (pHC210)	N/A
pET33b -His_6_-SUMO-Flag-PEX3ΔN-M72E	This paper (pHC211)	N/A
pET33b -His_6_-SUMO-Flag-PEX3ΔN-G138E	This paper (pHC52)	N/A
pET33b -His_6_-SUMO-Flag-PEX3ΔN-W104A	This paper (pHC95)	N/A
pET33b -His_6_-SUMO-Ram1	This paper (pHC40)	N/A
pET33b -His_6_-SUMO-Ram2	This paper (pHC41)	N/A
pET28a-2×Strep-SUMO-PEX26(237-305aa)	This paper (pHC46)	N/A
pET28a-2×Strep-SUMO-PEX26-L264^Amb^	This paper (pHC51)	N/A
tRNA_CUA_^Opt^ synthetase D286R	Young et al.^34^	N/A
pEGFP-2×Strep-PEX19-WT	This paper (pHC377)	N/A
pEGFP-2×Strep-PEX19-Δαa	This paper (pHC371)	N/A
pEGFP-2×Strep-PEX19-αd4A	This paper (pHC378)	N/A
pIRES2-GFP-PEX26(1-305aa); mCherry-SKL	This paper (pHC379)	N/A
pET33b -His_6_-TEV-SGTA	This paper (pHC426)	N/A

**Software and algorithms**		

iBright^TM^ analysis software	Thermo Fisher Scientific	https://www.thermofisher.com
pLink2 software (version 2.3.9)	Chen et al.^42^	http://pfind.org/software/pLink/index.html, RRID:SCR_000084
LAS X software (Version 4.6.1.27508)	Leica Microsystems	https://www.leica-microsystems.com/products/microscope-software/p/leica-las-x-ls/

**Other**		

OPTIZEN™ Alpha	KLAB	http://www.klabkis.com/english/0201_Alpha
UVP BLAK-RAY® B-100AP lamp	Analytik Jena	Cat#95-0127-02
100 W Bulb, Spot	Analytik Jena	Cat#34-0054-01
Amicon® Ultra 10,000 MWCO centrifugal filters	Millipore	Cat#UFC901024
iBrightFL1000 imaging system	Thermo Fisher Scientific	https://www.thermofisher.com
Leica Stellaris 8 confocal microscope	Leica	https://www.leica-microsystems.com/products/confocal-microscopes/p/stellaris-8/

### Resource availability

#### Lead contact

Further information and requests for resources and reagents should be directed to the lead contact, Hyunju Cho (hjcho@ibs.re.kr).

#### Materials availability

This study did not generate new unique reagents.

#### Data and code availability

• Data produced in this paper is available upon request to the lead contact.
• This paper does not report any original code.
• The mass spectrometry raw data have been deposited in Mendeley Data: https://doi.org/10.17632/dsrjx52zty.2 and are publicly available as of the date of publication.
• Any additional information required to reanalyze the data reported in this paper is available upon request to the lead contact.

### Experimental model and study participant details

#### Cell lines

HeLa and HEK293T cells were cultured in Dulbecco’s Modified Eagle Medium, GlutaMAXTM (DMEM, Gibco) supplemented with 10% (v/v) fetal bovine serum (FBS, Gibco), 100 U/mL streptomycin (Gibco), and 100 μg/mL penicillin (Gibco) and maintained in a humidified chamber with 5% CO_2_ at 37°C.

#### Microbe strains

E. coli DH5α (Enzynomics) or BL21 Star (DE3) (Invitrogen) competent cells were transformed and grown on plates of LB Agar with Carbenicillin or Kanamycin 50 μg/mL for plasmid selection at 37°C.

### Method details

#### Protein expression and purification

Expression of 2×Strep-SUMO-PEX26 (237-305aa) was induced with 0.1 mM isopropyl β-D-1-thiogalactopyranoside (IPTG) in BL21 Star (DE3) (Invitrogen) at 37°C for 1 h. Cells were resuspended in Buffer A (20 mM Tris-HCl (pH 8.0), 300 mM NaCl, 2 mM 2-Mercaptoethanol, 10% glycerol) supplemented with 125 U of benzonase (Sigma) and cOmplete EDTA-free protease inhibitor cocktail (Roche). Resuspended cells were incubated with 0.5% N,N-Dimethyl-1-Dodecanamine-N-Oxide (LDAO, Anatrace) and 1×CelLytic B Cell Lysis Reagent (Sigma) for 40 min at room temperature (25°C). The clarified lysate was then diluted 3-fold with Buffer A and loaded onto a Strep-Tactin Sepharose column (IBA Lifesciences). The resin was washed with Buffer A supplemented with 0.05% LDAO, after which the proteins were eluted with 15 mM *d*-Desthiobiotin (Sigma). Subsequently, the eluted proteins were dialyzed in Buffer B (20 mM HEPES (pH 7.5), 200 mM NaCl, 10% glycerol, 0.05% LDAO), concentrated with Amicon Ultra 10,000 MWCO centrifugal filters (Millipore), and stored at −80°C.

PEX19 (His_6_-PEX19-WT (1–299 aa), His_6_-PEX19-αd4A (F125A, L129A, L133A, L136A)), and HA-CaM (2–149 aa)-His_6_ were expressed in BL21 Star (DE3) with 0.5 mM IPTG at 18°C overnight. His_6_-SGTA (1–313 aa) and SUMO protease-His_6_ were expressed in BL21 Star (DE3) with 0.1 mM IPTG at 37°C for 4 h and 0.5 mM IPTG at 37°C for 2 h, respectively. The cells were resuspended in Buffer A and lysed by sonication. The clarified lysate was loaded onto Ni-NTA agarose resin (Qiagen) and then incubated at 4°C for 1 h. Proteins were eluted with 300 mM imidazole and dialyzed in Buffer C (20 mM HEPES (pH 7.5), 150 mM NaCl, 2 mM 2-Mercaptoethanol, 10% glycerol) and then stored at −80°C.

Expression of His_6_-SUMO-Flag-PEX3ΔN-WT (41-373aa), its variants (His_6_-SUMO-Flag-PEX3ΔN-M72K, His_6_-SUMO-Flag-PEX3ΔN-M72E, His_6_-SUMO-Flag-PEX3ΔN-G138E, His_6_-SUMO-Flag-PEX3ΔN-W104A), His_6_-SUMO-Ram1 (1–431 aa), and His_6_-SUMO-Ram2 (1–316 aa) was induced by the addition of 0.5 mM IPTG in BL21 Star (DE3), and the culture was incubated at 18°C overnight. The proteins were purified as described above. After the first Ni-NTA purification, the N-terminal His-SUMO tag was cleaved by SUMO-protease-His_6_ during the dialysis step. His_6_-SUMO and SUMO-protease-His_6_ were removed by performing an additional Ni-NTA purification. The flow-through fraction was collected, concentrated, and stored at −80°C.

To express 2×Strep-SUMO-PEX26^Bpa^, an amber codon (TAG) was introduced in the TMD-encoding sequence replacing the eighteenth amino acid (Leu264) (^247^FFSLPFKKSLLAALILCLLVV^267^). 2×Strep-SUMO-PEX26^Amb^ and tRNA_CUA_^Opt^ synthetase^34^ plasmids were co-transformed into BL21 Star (DE3) cells. The cells were grown to an optical density at 600 nm (OD_600_) of 0.3, and the expression of tRNA_CUA_^Opt^ synthetase was induced with 0.2% arabinose (Sigma). At OD_600_ of 0.6, expression of 2×Strep-SUMO-PEX26^Bpa^ was induced with 0.1 mM IPTG and 1 mM Bpa (Bachem) at 37°C for 1.5 h. His_6_-SUMO-PEX19^Bpa^ variants (F125^Bpa^, L129^Bpa^, L133^Bpa^, L136^Bpa^, and M179^Bpa^) were expressed with a similar method as 2×Strep-SUMO-PEX26^Bpa^, apart from the IPTG concentration, which was 0.5 mM. All proteins were purified in the same way as their non-Bpa proteins.

#### *In vitro* farnesylation reaction

*In vitro* farnesylation reaction was carried out as described previously, with a minor modification.^10^ 30 μM His_6_-PEX19 was incubated with 700 nM farnesyl transferases, Ram1 and Ram2, and 65 μM farnesyl pyrophosphate (Sigma) in the reaction buffer (50 mM Tris-HCl (pH 8.0), 20 mM CH_3_COOK, 5 mM MgCl_2_, 0.1 mM ZnCl_2_, and 10 mM DTT) at room temperature for 1 h. The reaction mixture was diluted 10-fold with Buffer C and then incubated with Ni-NTA agarose resin for 5 min. The farnesylated PEX19 proteins were purified using a standard Ni-NTA purification method.

#### Limited proteolysis of PEX19 proteins

20 μM PEX19 was incubated with 1.5 μg/mL trypsin (Sigma) at 37°C. At the indicated time, 12 μL of samples were mixed with SDS loading buffer and heated at 95°C for 5 min. Subsequently, 5 μL of quenched samples were loaded onto 12.5% Tris-glycine gels. The gels were stained with SimplyBlue SafeStain (Invitrogen).

#### Turbidity assay

200 μM PEX26 stored in Buffer B containing 0.05% LDAO was rapidly diluted to a final concentration of 1.5 μM in the assay buffer (20 mM K-HEPES (pH 7.5), 150 mM CH_3_COOK, 1 mM DTT) containing various concentrations of PEX19 proteins. The optical density of PEX26 at 360 nm was measured in real time using a UV spectrophotometer (Optizen Alpha, KLAB). The observed solubility of PEX26 (S_*obsd*_) was calculated from the % change of the OD_360_ value at 5 min between PEX26 alone and in the PEX19-containing samples. The data were plotted as a function of PEX19 concentration and fit to Equation 1,

(Equation 1) $$S_{obsd}=S_{\mathrm{Max}}\times \frac{\left[PEX19\right]}{K_{soluble}+\left[PEX19\right]}$$

in which S_*Max*_ is the % soluble PEX26 at saturating PEX19 concentrations, and *K*_*soluble*_ is the concentration of PEX19 required to reach half of S_*Max*_.

#### Preparation of PEX19·PEX26^Bpa^ complex

6 μM His_6_-PEX19 and 1.5 μM PEX26^Bpa^ were incubated in the pulldown assay buffer (20 mM K-HEPES (pH 7.5), 150 mM CH_3_COOK, 2 mM 2-Mercaptoethanol, 10% glycerol) at room temperature for 5 min. The reaction mixture was subsequently incubated with Talon resin (Takara) at 4°C for 10 min. After washing the resin with the pulldown assay buffer, the PEX19·PEX26^Bpa^ complexes were eluted with 200 mM Imidazole. The purified complexes were buffer-exchanged, aliquoted, and flash-frozen in liquid nitrogen.

#### Bpa crosslinking assay to monitor the dissociation of PEX26 from PEX19

PEX19·PEX26^Bpa^ (400 nM at final concentration of PEX19) complex was incubated with 20 μM CaM in the assay buffer (20 mM K-HEPES (pH 7.5), 150 mM CH_3_COOK, 1 mM Ca(C_2_H_3_O_2_)_2_, 1 mM DTT, 10% glycerol) at room temperature. At the indicated time points, 15 μL of samples were flash-frozen in liquid nitrogen. The frozen samples were placed on dry ice and irradiated at ∼4 cm from a UVP BLAK-RAY B-100AP lamp (Analytik Jena) for 10 min. The total crosslinked samples (15 μL) were loaded onto 8% Tricine gel and detected by western blotting using Strep (1:3000 dilution, Genscript) and IRDye800 secondary antibodies (1:20,000 dilution, LiCor). The non-crosslinked and crosslinked bands of PEX26 were detected using iBrightFL1000 imaging system (Thermo Fisher Scientific), and their intensities were quantified using iBright analysis software (Thermo Fisher Scientific). PEX26 dissociation efficiency was calculated as [I_PEX26xCaM_]/[I_PEX26xPEX19_ + I_PEX26xCaM_]∗100, where I denotes the intensity of the band of interest. Given that a longer incubation time and mutation of the αd helix of PEX19 could lead to non-specific interactions between the hydrophobic PEX26^Bpa^ and the plastic surfaces, we only calculated the relative crosslinked fraction of PEX26 within each sample. PEX26 dissociation efficiency data in the presence of various concentrations of CaM were fit to Equation 2,

(Equation 2) $$PEX26\;dissociation\;\left(\%\right)=A\left(1-e^{-kt}\right)$$

in which *k* is the rate constant in the dissociation reaction, and A is the amplitude of the single phase.

#### Bpa crosslinking assay to monitor the PEX19^Bpa^ interaction with PEX3ΔN

400 nM PEX19^Bpa^ was incubated without and with 100 nM PEX26 at room temperature for 1 min. The reaction was further incubated with 400 nM PEX3ΔN-WT and its variants at room temperature for 5 min. The reaction was UV-irradiated as described in the PEX26^Bpa^ crosslinking assay. The crosslinked samples were loaded onto 8% Tris-glycine gels and analyzed by western blotting using Strep (1:3000 dilution, Genscript), PEX19 (1:3000 dilution, Novus Biologicals), Flag (1:3000 dilution, Genscript), and IRDye800 secondary antibodies (1:20,000 dilution, LiCor).

#### LC-MS/MS crosslinking analysis

PEX19-F125^Bpa^ and PEX3ΔN-WT at the final concentration of 10 μM were incubated in the buffer (20 mM K-HEPES (pH 7.5), 150 mM CH_3_COOK, 10% glycerol) at room temperature for 5 min. The reaction was UV-irradiated as described in the Bpa crosslinking assay. The PEX19-F125xPEX3ΔN-WT crosslinked band was resolved by SDS-PAGE and then stained with SimplyBlue SafeStain (Invitrogen). The excised gel piece was further digested with trypsin (Sigma) and AspN (Roche). The digested peptides were extracted using the extraction buffer (75% acetonitrile and 0.1% TFA),^53^ and analyzed by LTQ Orbitrap XL system (Thermo Fisher Scientific) from KBSI (Korea Basic Science Institute) MS analysis service.

MS raw data were searched using pLink2 software (version 2.3.9),^42^ with three missed cleavages, carbamidomethylation of cysteines as a fixed modification, and oxidation of methionines as a variable modification. Peptide mass was set to 400 to 10,000 Da, and peptide length of amino acid was set to 4 to 40 residues. The precursor tolerance and the fragment tolerance were 10 ppm and 20 ppm, respectively. The results were filtered by a false discovery rate of 5% and a precursor mass accuracy of ±10 ppm.

#### Cell culture and DNA transfection

HeLa and HEK293T cells were cultured in Dulbecco’s Modified Eagle Medium, GlutaMAX (DMEM, Gibco), supplemented with 10% (v/v) FBS (Gibco), 100 U/mL streptomycin (Gibco), and 100 μg/mL penicillin (Gibco) in a humidified chamber with 5% CO_2_ at 37°C. For the co-expression of 2×Strep-PEX19 variants and GFP-PEX26, 3 × 10^5^ cells per well were seeded on a 6-well plate one day prior to the transfection. Transient transfections of plasmids (0.75 μg each) were carried out with Lipofectamine 3000 (Invitrogen) according to the manufacturer’s instructions.

#### Preparation of the 2×Strep-PEX19·GFP-PEX26 complex from HEK293T cytosol fraction

2×Strep-PEX19·GFP-PEX26 complex was prepared using a standard Strep pulldown assay.^17^ HEK293T cells co-transfected with plasmids containing 2×Strep-PEX19 variants and GFP-PEX26 were washed with DPBS twice and then incubated with SP buffer (20 mM HEPES (pH 7.4), 25 mM KCl, 1 mM DTT, 0.25 M Sucrose, 2.5 mM EGTA, 2.5 mM Mg(CH₃COO)₂) containing 100 μg/mL of digitonin (Sigma) and 1× Halt protease inhibitor cocktail (Thermo Fisher Scientific) for 10 min on ice. After centrifugation for 15 min at 20,000 g, the cytosolic fraction (supernatant) was collected and incubated with Strep-Tactin Sepharose resin at 4°C for 30 min. The column was washed with SP buffer, and then the proteins were eluted with 15 mM d-Desthiobiotin dissolved in the same SP buffer. The 2×Strep-PEX19·GFP-PEX26 complexes were buffer-exchanged with SP buffer and then immediately used for PEX26 import assay into semi-permeabilized HeLa cells.

#### *In vitro* PEX26 import assay using semi-permeabilized HeLa cells

HeLa cells cultured on glass coverslips were washed with ice-cold SP buffer and then incubated with SP buffer supplemented with 50 μg/mL digitonin and 1× Halt protease inhibitor cocktail for 10 min. The semi-permeabilized HeLa cells were washed twice with SP buffer to minimize residual proteins in the cytoplasm. In the absence and presence of 3 μM PEX3ΔN-WT, 2×Strep-PEX19·GFP-PEX26 complexes prepared from the HEK293T cell cytosolic fraction were incubated with the semi-permeabilized cells at room temperature for 1 h. After an extensive wash with SP buffer, the cells were fixed with 4% paraformaldehyde for 10 min for immunofluorescence.

To stain GFP-PEX26 and PMP70, the fixed cells were further permeabilized using 0.1% Triton X-100 in PBS for 5 min, followed by blocking with 2% BSA in PBS. The cells were incubated with GFP (1:250 dilution, Abcam) and PMP70 (1:200 dilution, Invitrogen) primary antibodies overnight at 4°C. The cells were then washed with PBS and further incubated with Alexa Fluor 568-conjugated goat anti-rabbit secondary antibody (1:1000 dilution, Invitrogen) or Alexa 647-conjugated fragment donkey anti-chicken secondary antibody (1:1000 dilution, Jackson ImmunoResearch) for 1 h at room temperature. For nuclear staining, the cells were treated with DAPI (Invitrogen) for 10 min. After washing with PBS, coverslips were mounted onto slide glass with PBS. High-resolution images were acquired on Leica Stellaris 8 confocal microscope using a 63x/1.4 oil objective under LIGHTNING mode. The mean intensities and rates of PEX26 colocalization with the peroxisomal marker protein (PMP70) were analyzed by LAS X software (Version 4.6.1.27508). The colocalization rates (%) were calculated from the ratio of the area of colocalizing fluorescence signals (Colocalization Area) and the area of the image foreground (Area Foreground).

#### PEX19-F125^Bpa^ crosslinking using semi-permeabilized HeLa cells

HeLa cells on the culture dish were semi-permeabilized, as described above. To remove cytosolic proteins, the cells were extensively washed with SP buffer. The semi-permeabilized HeLa cells were incubated with 200 nM PEX19-F125^Bpa^ protein at room temperature for 1 h. The cells were irradiated at ∼4 cm from a UVP BLAK-RAY B-100AP lamp (Analytik Jena) for 10 min on ice. After UV crosslinking, the cells were scraped and lysed by the incubation in the lysis buffer (50 mM Tris-HCl (pH 7.4), 100 mM NaCl, 1 mM MgCl_2_, 0.5% SDS, 0.15 U/μl benzonase) supplemented with 1× Halt protease inhibitor cocktail for 30 min on ice. After adding 1.5% SDS and 50 mM DTT, the crosslinked cell lysate was boiled at 95°C for 10 min, loaded onto 10% Tris-Glycine gel, and analyzed by western blotting using PEX19 (1:3000 dilution, Novus Biologicals) and PEX3 (1:3000 dilution, Novus Biologicals) primary antibodies and IRDye800 secondary antibody (1:20,000 dilution, LiCor).

### Quantification and statistical analysis

The data of colocalization rate are presented as the mean ± SD. Two-tailed Student’s *t* test was performed to evaluate whether the difference between two groups is statistically significant. A p value of <0.05 was considered statistically significant.
