# Supplementary material for: A dual role of the conserved PEX19 helix in safeguarding peroxisomal membrane proteins
Source: iScience. 2024 Mar 18;27(4):109537. doi: 10.1016/j.isci.2024.109537 (PMC10995880; doi:10.1016/j.isci.2024.109537)
Supplement: Document S1. Figures S1–S6 and Table S1 [file mmc1.pdf]

iScience, Volume *27*

Supplemental information

A dual role of the conserved PEX19 helix in safeguarding peroxisomal membrane proteins

Jeonghyun Oh, Do Kyung Kim, Seung Hae Ahn, Ho Min Kim, and Hyunju Cho

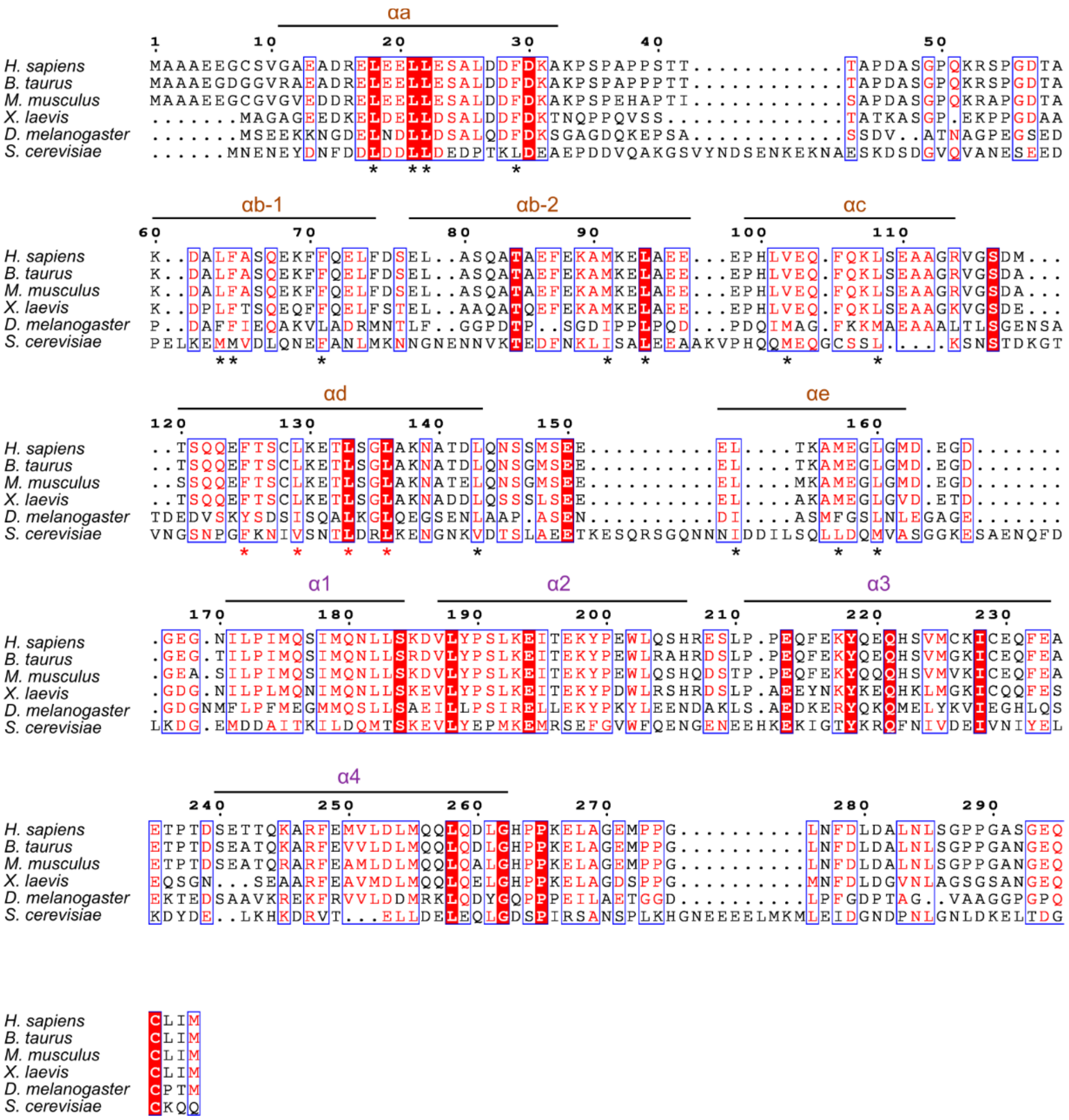

Figure S1. A multiple sequence alignment of PEX19 proteins, Related to Figure 1

A multiple sequence alignment of the PEX19 sequences from *Homo sapiens* (Uniprot code P40855), *Bos taurus* (Q3SZD1), *Mus musculus* (Q8VCI5), *Xenopus laevis* (Q0IH62), *Drosophila melanogaster* (Q8IP97), and *Saccharomyces cerevisiae* (Q07418) was performed using Clustal Omega[1], and the alignment was visualized using ESPript 3[2]. The alpha helices of PEX19-NTD and PEX19-CTD were assigned based on the AlphaFold predicted structure (AF-P40855-F) and the NMR structure (PDB 5LNF)[3], respectively. All conserved hydrophobic residues in the PEX19-NTD are marked with an asterisk (*). As also shown in the previous study[4], the hydrophobic residues in the αd helix of PEX19 were highly conserved among tested species, and the mutated residues in PEX19-αd4A are highlighted in red.

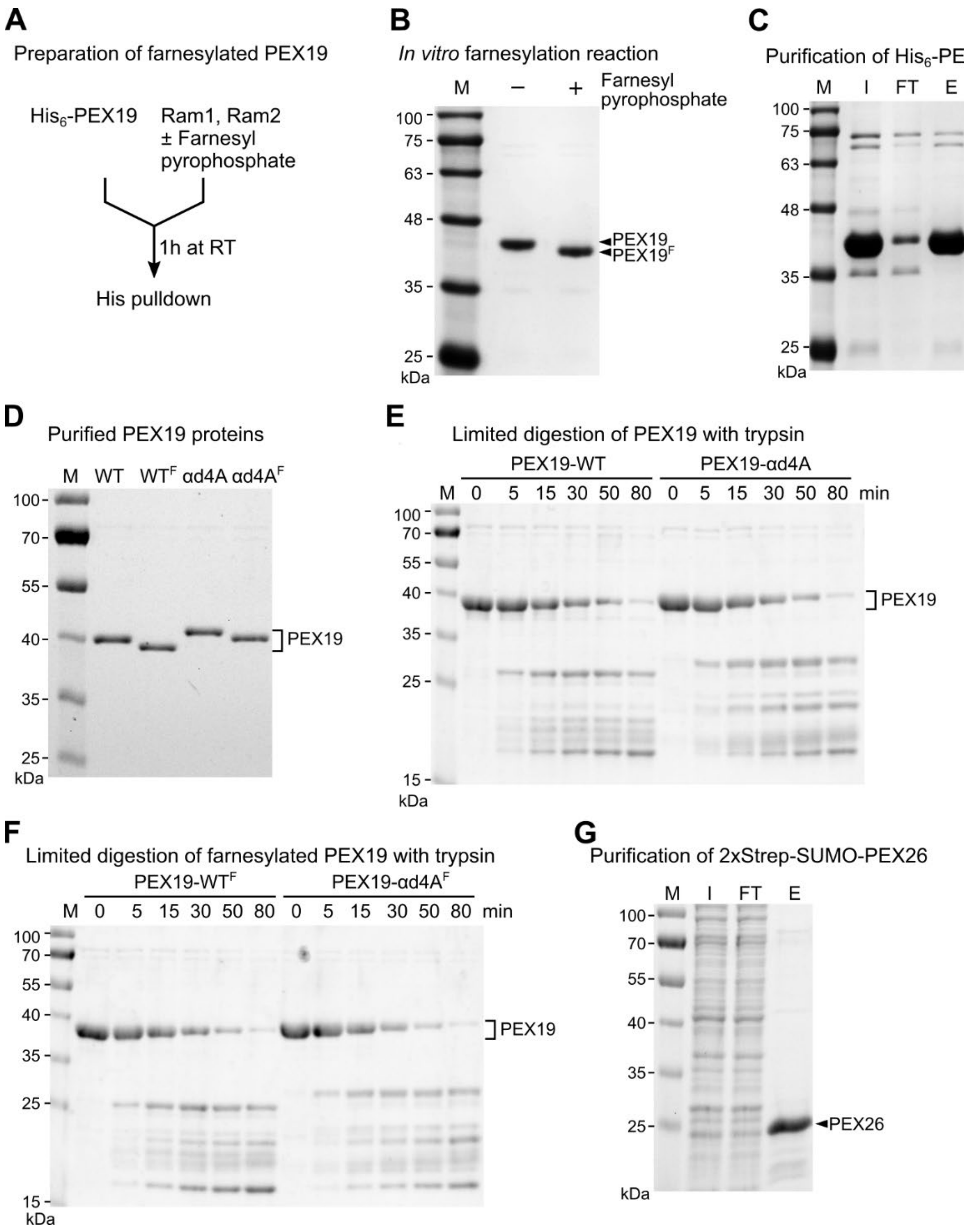

Figure S2. Preparation of recombinant proteins, Related to Figure 1

(A) A schematic representation of the *in vitro* farnesylation assay used to prepare the farnesylated PEX19 protein. After incubating His_6-PEX19 with Hisless farnesyl transferases (Ram1 and Ram2) and farnesyl pyrophosphate, the farnesylated His_6-PEX19 protein was affinity-purified using Ni-NTA resin.

(B) *In vitro* farnesylation reaction. The PEX19 protein was fully farnesylated in the presence of farnesyl pyrophosphate, as evident from a complete shift of the PEX19 band in the SDS-PAGE gel.

(C and D) Purification of farnesylated PEX19 proteins. (C) Hisless farnesyl transferases and unreacted farnesyl pyrophosphate were removed during the His_6-tag purification. The input (I), flow-through (FT), and elution (E) fractions were resolved by SDS–PAGE and visualized by Coomassie staining. (D) 3 μM PEX19-WT, farnesylated PEX19-WT^F, PEX19-αd4A, and farnesylated PEX19-αd4A^F proteins (5 μL) were loaded onto a 10% Tris-glycine gel. Both PEX19-WT^F and PEX19-αd4A^F proteins migrated faster than their corresponding non-farnesylated proteins.

(E and F) Limited proteolysis of PEX19 proteins using trypsin. 20 μM non-farnesylated PEX19 (E) and farnesylated PEX19 (F) proteins were incubated with 1.5 μg/mL trypsin at 37 °C. At the indicated time, samples were mixed with 1× SDS loading buffer and heated at 95 °C for 5 min. The samples were resolved on 12.5% Tris-glycine gels and subsequently stained using SimplyBlue™ SafeStain. Since PEX19-αd4A proteins migrated slower than PEX19-WT proteins, as shown in (D), the digested fragments containing αd4A also ran slower compared to the WT proteins.

(G) Purification of PEX26. 2×Strep-SUMO-PEX26 protein was detergent-purified using Strep-Tactin resin.

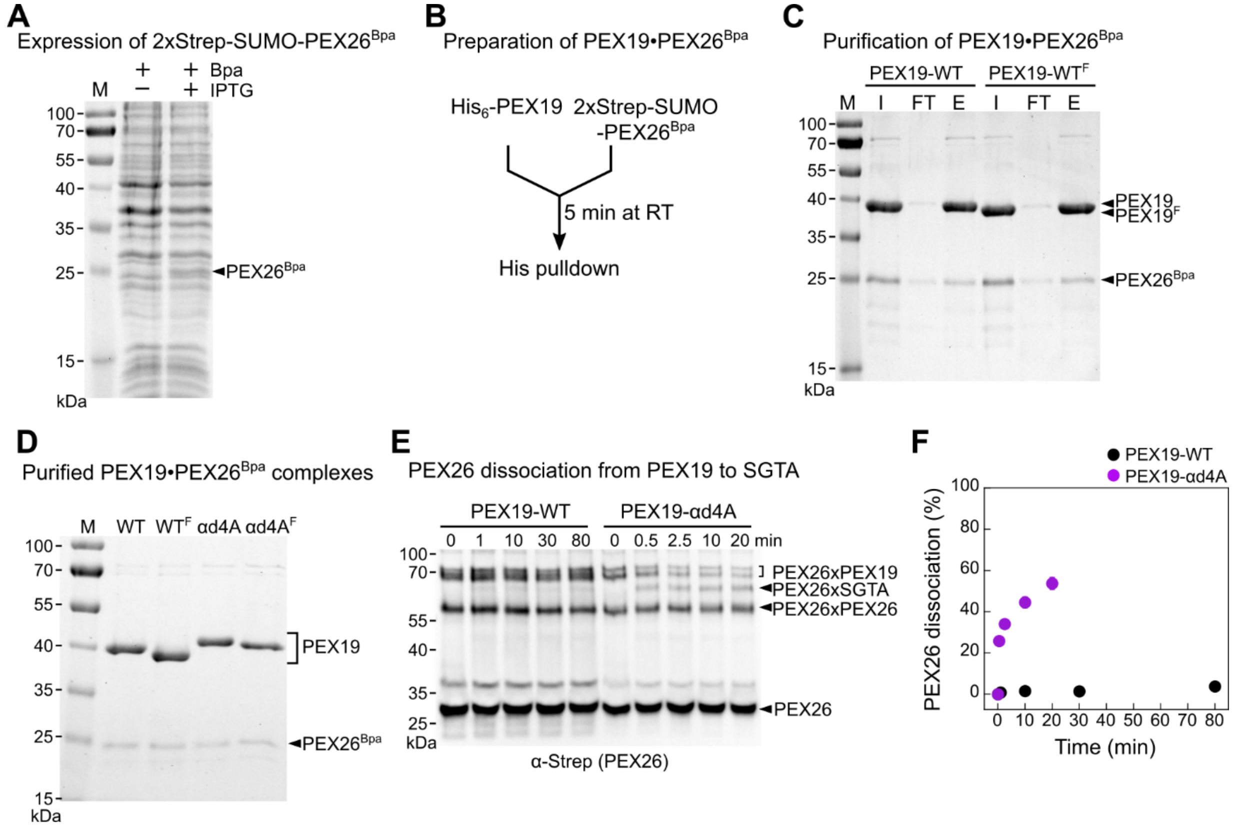

Figure S3. The αd helix of PEX19 prevents PEX26 loss to SGTA, Related to Figure 1

(A) Expression of $PEX26^{Bpa}$. After induction with 0.1 mM IPTG, the Bpa-incorporated 2×Strep-SUMO-PEX26 protein was expressed. The purification of $PEX26^{Bpa}$ was carried out as described in STAR Methods.

(B) A schematic representation of the preparation of PEX19•$PEX26^{Bpa}$ complexes. 1.5 µM of 2×Strep-SUMO-$PEX26^{Bpa}$ was incubated with 6 µM of His_6-PEX19 at room temperature for 5 min, and the resulting PEX19•$PEX26^{Bpa}$ complexes were affinity-purified using Talon resin.

(C and D) Purification of the PEX19•$PEX26^{Bpa}$ complexes. The levels of $PEX26^{Bpa}$ associated with PEX19 variants in the purified complexes are comparable.

(E and F) The dissociation of PEX26 from PEX19 to SGTA. The release of PEX26 from PEX19 to SGTA was monitored using the Bpa crosslinking assay outlined in Figure 1H. The PEX19•$PEX26^{Bpa}$ complex was incubated with 30 µM SGTA, after which the samples were freshly frozen at the indicated time. After UV crosslinking, the samples were resolved on an 8%

Tricine gel, and the crosslinked products were detected using a Strep antibody in (E). PEX26 dissociation from the Bpa crosslinking data in (E) and their replicates were quantified in (F) as described in STAR Methods. All values in (E) are reported as mean ± SEM, with n=2. Error bars are shown but may not be visible in most cases.

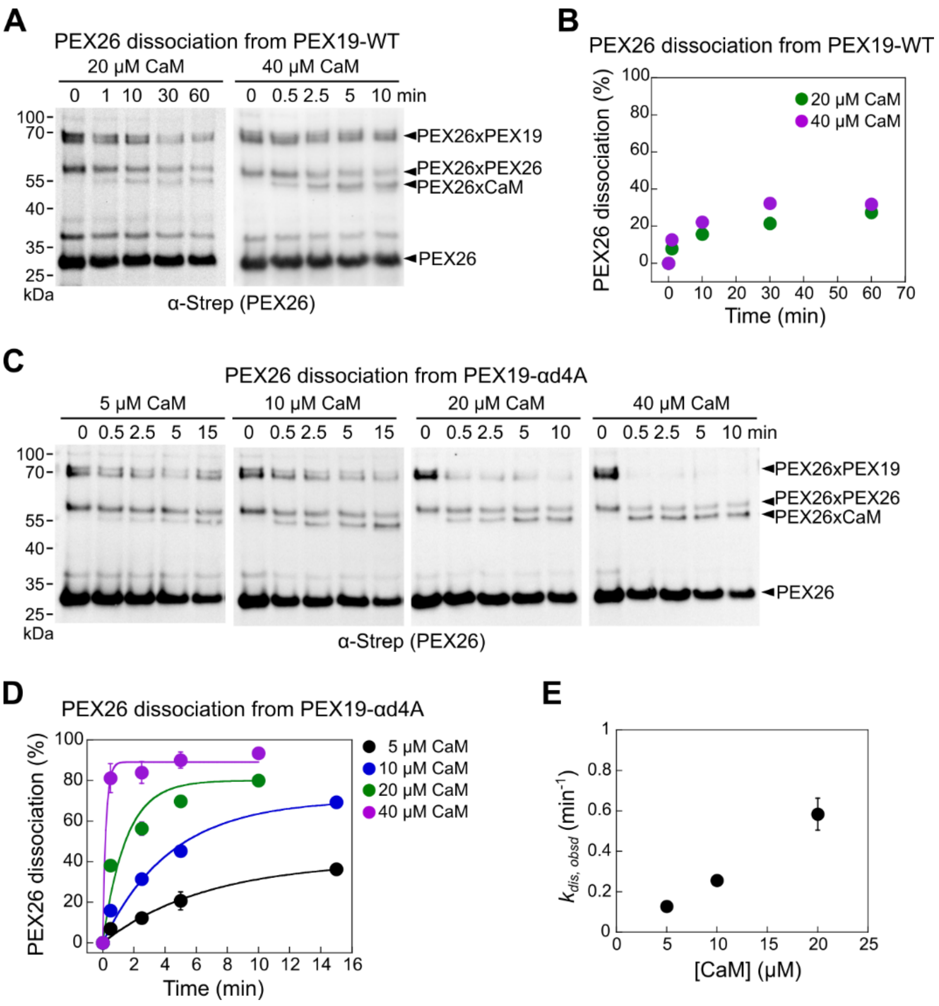

Figure S4. The αd helix of PEX19 prevents the release of PEX26 from PEX19 to off-pathway chaperones, Related to Figure 1

(A and B) (A) A representative western blot image of PEX26^{Bpa} dissociation from PEX19-WT in the presence of CaM as a chase. (B) The quantification of PEX26 dissociation from the Bpa crosslinking data in (A) and their replicates.

(C and D) (A) A representative western blot image of PEX26^{Bpa} dissociation from PEX19-αd4A in the presence of CaM. (D) The quantification of PEX26 dissociation from the Bpa crosslinking data in (C) and their replicates.

(E) The observed dissociation rates of PEX26^{Bpa} from PEX19-αd4A. The data fit Equation 2 as described in STAR Methods. Increasing concentrations of CaM substantially increased the observed rates of TA loss from PEX19-αd4A.

All values in (B), (D), and (E) are reported as mean ± SEM, with n=2. Error bars are shown but may not be visible in some cases.

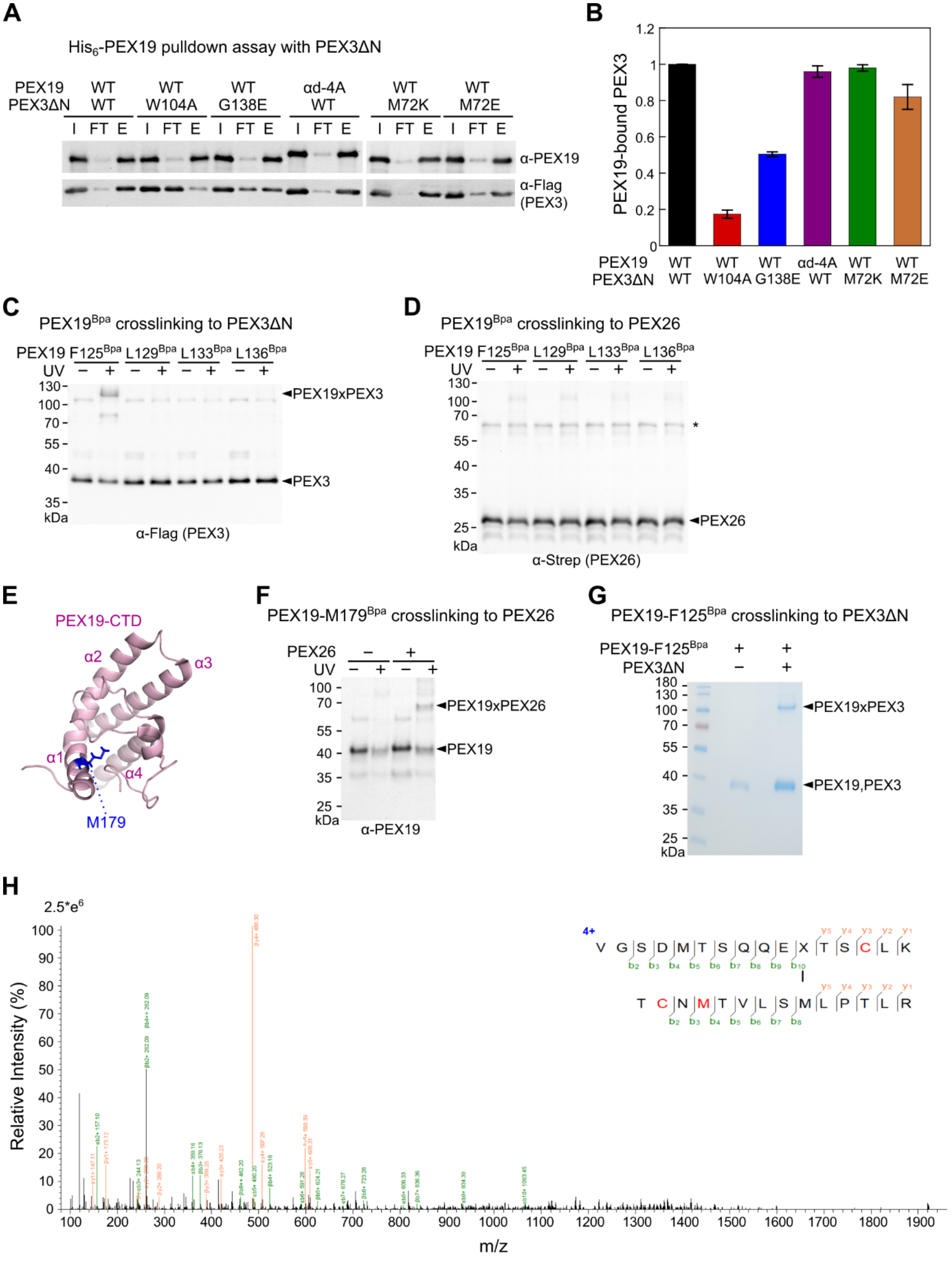

A
His6-PEX19 pulldown assay with PEX3ΔN
PEX19 WT WT αd-4A WT WT WT
PEX3ΔN WT W104A G138E WT M72K M72E
I FT E
α-PEX19
α-Flag (PEX3)
B
PEX19-bound PEX3
PEX19 WT WT WT αd-4A WT WT
PEX3ΔN WT W104A G138E WT M72K M72E
C
PEX19Bpa crosslinking to PEX3ΔN
PEX19 F125Bpa L129Bpa L133Bpa L136Bpa
UV − + − + − + − +
PEX19xPEX3
PEX3
kDa
α-Flag (PEX3)
D
PEX19Bpa crosslinking to PEX26
PEX19 F125Bpa L129Bpa L133Bpa L136Bpa
UV − + − + − + − +
*
PEX26
kDa
α-Strep (PEX26)
E
PEX19-CTD
α1
α2
α3
α4
M179
F
PEX19-M179Bpa crosslinking to PEX26
PEX26 − +
UV − + − +
PEX19xPEX26
PEX19
kDa
α-PEX19
G
PEX19-F125Bpa crosslinking to PEX3ΔN
PEX19-F125Bpa + +
PEX3ΔN − +
PEX19xPEX3
PEX19,PEX3
kDa
H
2.5*e6
Relative Intensity (%)
m/z
4+
V G S D M T S Q Q E X T S C L K
T C N M T V L S M L P T L R

Figure S5. The αd helix of PEX19 serves as a secondary PEX3-binding site, Related to Figure 3

(A) A representative western blot image of the His_6-PEX19 pulldown assay with PEX3ΔN. 400 nM His_6-PEX19 was incubated with 400 nM PEX3ΔN at room temperature for 5 min. The reaction was subjected to affinity purification using Talon resin.

(B) The quantification of PEX19-bound PEX3ΔN from the data in (A) and replicates. Values are reported as mean ± SEM, with n=2.

(C and D) (C) $PEX19^{Bpa}$ crosslinking to PEX3ΔN-WT. Among the four hydrophobic amino acids (F125, L129, L133, L136) in the αd helix of PEX19, only PEX19-$F125^{Bpa}$ crosslinked with PEX3ΔN-WT. (D) In contrast, none of these hydrophobic amino acids crosslinked with PEX26. In (D), the SDS-resistant PEX26 dimer was denoted by the symbol "*".

(E) The NMR structure of PEX19-CTD (PDB 5LNF)[3]. Bpa was incorporated at the M179 residue located in the PMP binding site, α1.

(F) Western blot analysis of PEX19-$M179^{Bpa}$ crosslinking to PEX26. The crosslinked proteins were probed using an anti-PEX19 antibody.

(G) An SDS-PAGE gel image depicting the PEX19-$F125^{Bpa}$-PEX3ΔN-WT crosslink. The band corresponding to the crosslinked protein complex was excised and analyzed by mass spectrometry.

(H) A representative MS/MS spectrum of one of the PEX19-$F125^{Bpa}$-PEX3ΔN-WT crosslinked peptides. "X" denotes the Bpa residue in the PEX19 peptide (115-130 aa). The M72 residue of PEX3 was crosslinked with the $F125^{Bpa}$ of PEX19.

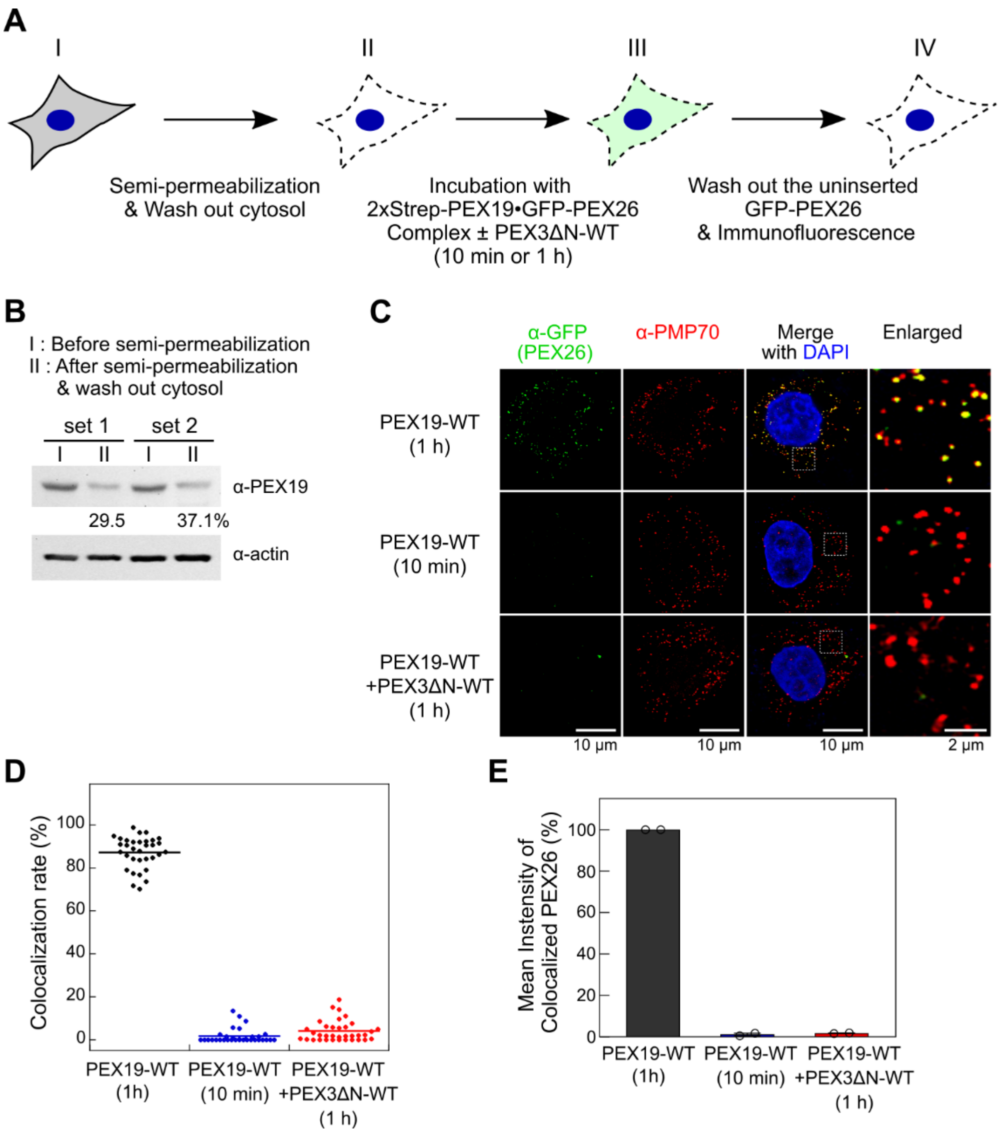

Figure S6. Trapping the PEX19-PEX26 complex with PEX3ΔN in the cytosol abolished PEX26 localization to the peroxisome, Related to Figure 4

(A) A schematic representation of the *in vitro* PEX26 import assay into the peroxisome. HeLa cells (stage I) were semi-permeabilized, and the cytosolic proteins were washed out. The semi-permeabilized HeLa cells (stage II) were incubated with freshly prepared 2×Strep-PEX19•GFP-PEX26 complexes for either 10 min or 1 h, both in the absence and presence of PEX3ΔN-WT. The cells (stage III) were washed to remove non-targeted complexes and then processed for immunofluorescence analysis (stage IV).

(B) Western blot analysis of endogenous PEX19 before and after semi-permeabilization. Total cell lysates were separated by SDS-PAGE and immunoblotted with anti-PEX19 and anti-actin antibodies. The semi-permeabilized HeLa cells may contain approximately one-third of cytosolic proteins.

(C) Immunofluorescence analysis of PEX26 localization in the semi-permeabilized HeLa cells. The 2×Strep-PEX19•GFP-PEX26 complexes were incubated with the semi-permeabilized cells without or with PEX3ΔN-WT. After the indicated times, the cells were washed, fixed, and analyzed by immunofluorescence (Scale bar: 10 μm). The right panel displays 5.3-fold enlarged images of the boxed areas (Scale bar: 2 μm).

(D) Colocalization rates of PEX26 with the peroxisomal membrane protein (PMP70). A total of 30~33 cells from two biological replicates were analyzed using LAS X software. The lines indicate the mean values for each condition.

(E) Mean intensity of colocalized PEX26 with PMP70. The mean intensities of peroxisome-localized PEX26 from the same cells in (D) were analyzed using LAS X software. Values are reported as mean ± SEM, with n=2. Error bars are shown but may not be visible in some cases.

Table S1. A list of $K_{soluble}$ values reported in Figure 1G, Related to Figure 1

Listed below are the PEX19 concentrations required for half-maximal solubilization of PEX26 ($K_{soluble}$). The data in Figure 1B-1E and their replicates were fitted to Equation 1. Each data fitting generates fitting errors. The mean and SD values for $K_{soluble}$ were also reported in Figure 1G (n=3).

	$K_{soluble}$ (μM)	Fitting error (μM)	Mean ± SD (μM)
PEX19-WT	1.95	± 0.17	
	1.58	± 0.08	1.71 ± 0.21
	1.59	± 0.07	
PEX19-αd4A	3.04	± 0.30	
	3.18	± 0.32	2.96 ± 0.27
	2.67	± 0.24	
PEX19-WT^F	0.73	± 0.04	
	0.67	± 0.06	0.72 ± 0.04
	0.74	± 0.06	
PEX19-αd4A^F	1.12	± 0.08	
	1.13	± 0.06	1.16 ± 0.07
	1.24	± 0.12	

Supplementary references

1. Sievers, F., Wilm, A., Dineen, D., Gibson, T.J., Karplus, K., Li, W., Lopez, R., McWilliam, H., Remmert, M., Söding, J., et al. (2011). Fast, scalable generation of high-quality protein multiple sequence alignments using Clustal Omega. Mol. Syst. Biol. *7*, 539. 10.1038/msb.2011.75.

2. Robert, X., and Gouet, P. (2014). Deciphering key features in protein structures with the new ENDscript server. Nucleic Acids Res. *42*, W320-324. 10.1093/nar/gku316.

3. Emmanouilidis, L., Schütz, U., Tripsianes, K., Madl, T., Radke, J., Rucktäschel, R., Wilmanns, M., Schliebs, W., Erdmann, R., and Sattler, M. (2017). Allosteric modulation of peroxisomal membrane protein recognition by farnesylation of the peroxisomal import receptor PEX19. Nat. Commun. *8*, 14635. 10.1038/ncomms14635.

4. Chen, Y., Pieuchot, L., Loh, R.A., Yang, J., Kari, T.M.A., Wong, J.Y., and Jedd, G. (2014). Hydrophobic handoff for direct delivery of peroxisome tail-anchored proteins. Nat. Commun. *5*, 5790. 10.1038/ncomms6790.
